# Supplementary material for: Single-cell insights into immune dysregulation in rheumatoid arthritis flare versus drug-free remission
Source: Nat Commun. 2024 Feb 5;15:1063. doi: 10.1038/s41467-024-45213-2 (PMC10844292; doi:10.1038/s41467-024-45213-2)
Supplement: Supplementary file 1 — Supplementary Information [file 41467_2024_45213_MOESM1_ESM.pdf]

Supplementary Information for

Single-cell insights into immune dysregulation in rheumatoid arthritis flare versus drug-free remission

Kenneth F. Baker *et al.*

Corresponding author: Kenneth F Baker, kenneth.baker@ncl.ac.uk

The PDF file includes:

Supplementary Figures 1 to 17

Supplementary Tables 1 to 4

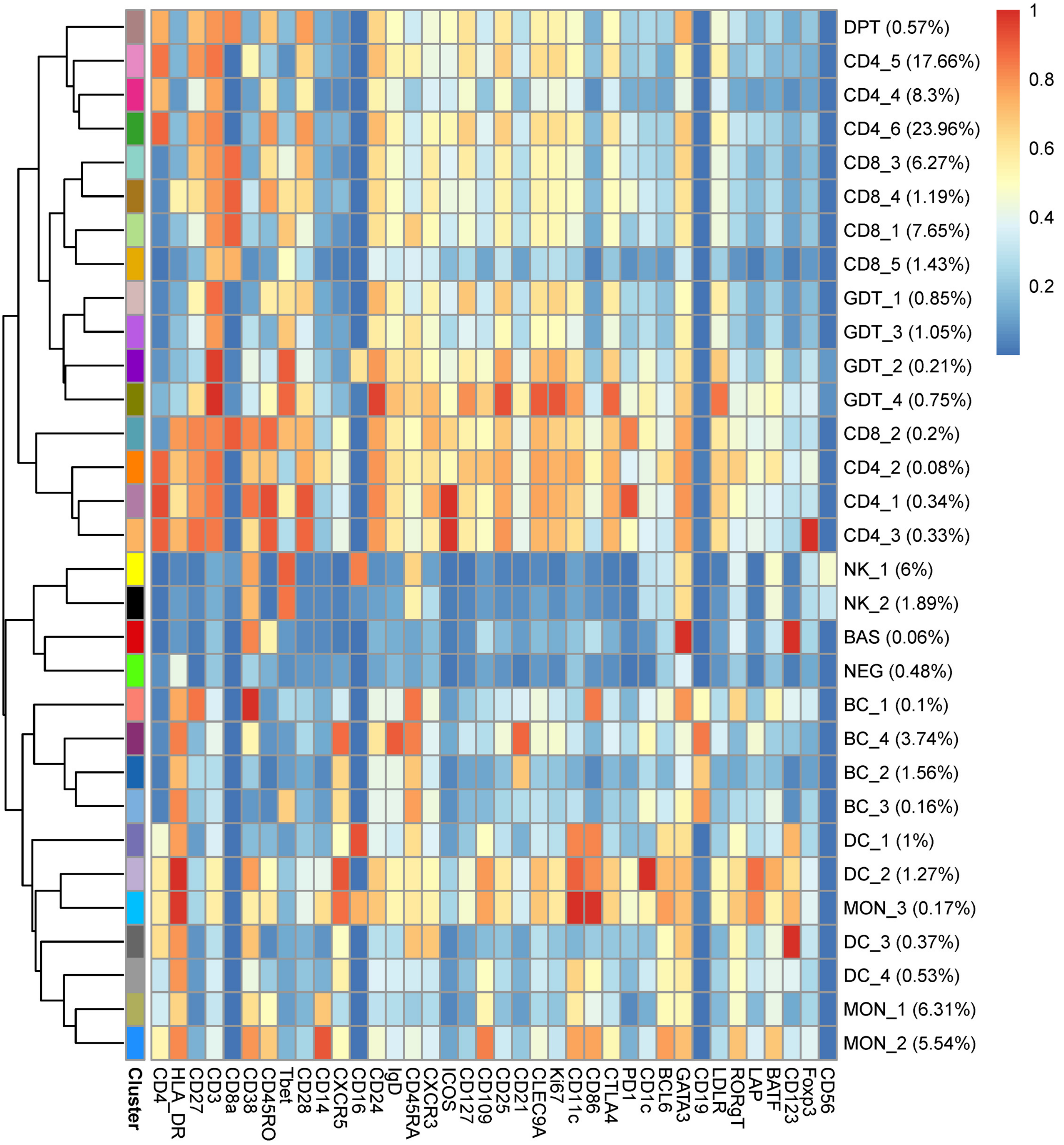

Supplementary Figure 1. **Heatmap showing marker expression across mass cytometry clusters.** Colours denote median arcsinh transformed (0-1) marker expression (columns) for all patients across different clusters (rows). The dendrogram shows hierarchical clustering by Euclidean distance with average linkage.

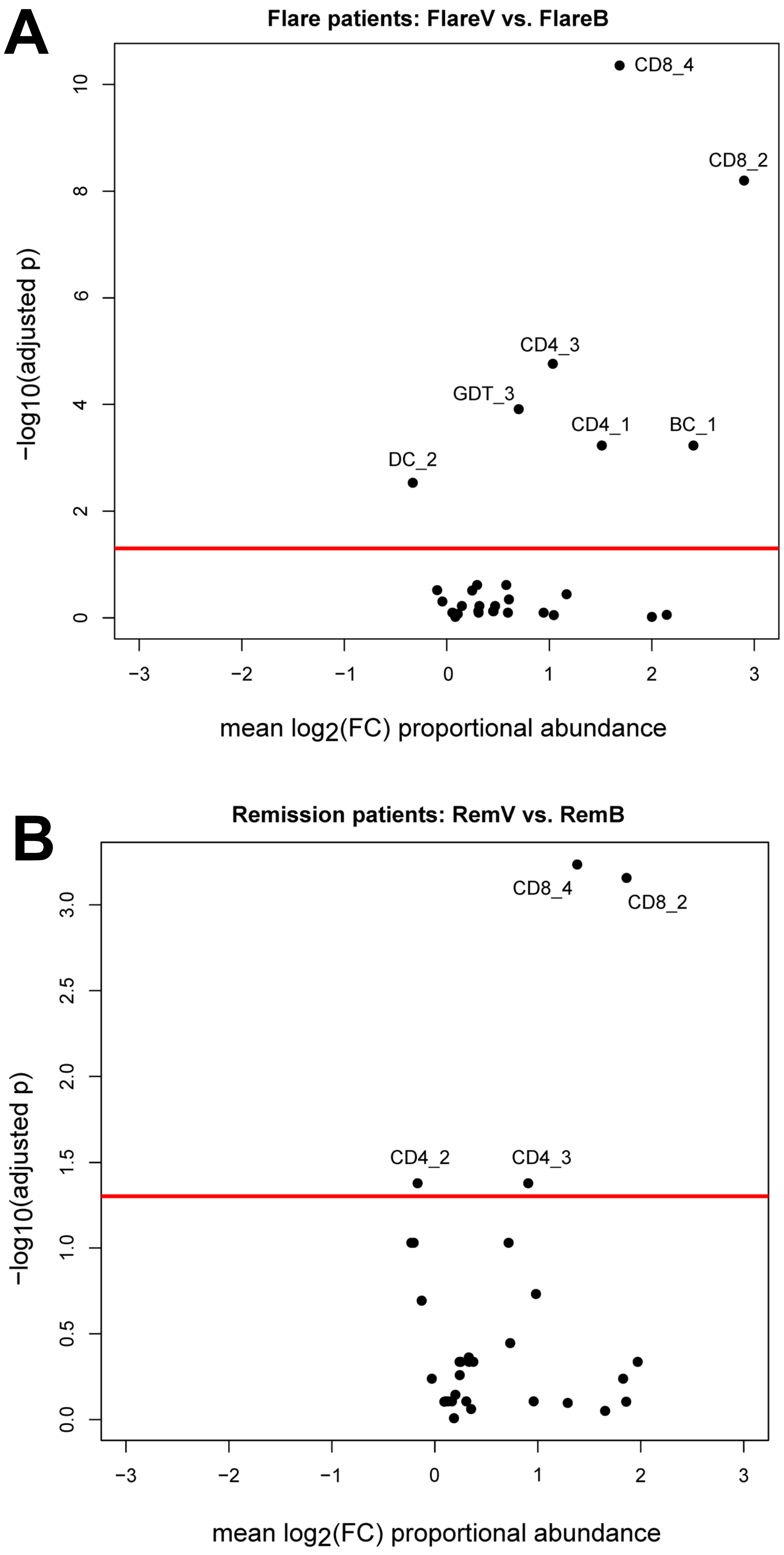

Supplementary Figure 2. **Volcano plots showing longitudinal change in proportional abundance of PBMC clusters.** A: flare patients (n=20:flare vs baseline visits), B: DFR patients (n=16: month 6 vs baseline visits). Red line shows adjusted two-sided p<0.05 threshold (generalised linear mixed model, Benjamini-Hochberg correction). FlareB: flare patient, baseline visit; FlareV: flare patient, flare visit; RemB: remission patient, baseline visit; RemV: remission patient, month 6 visit. Source data are provided as a Source Data file.

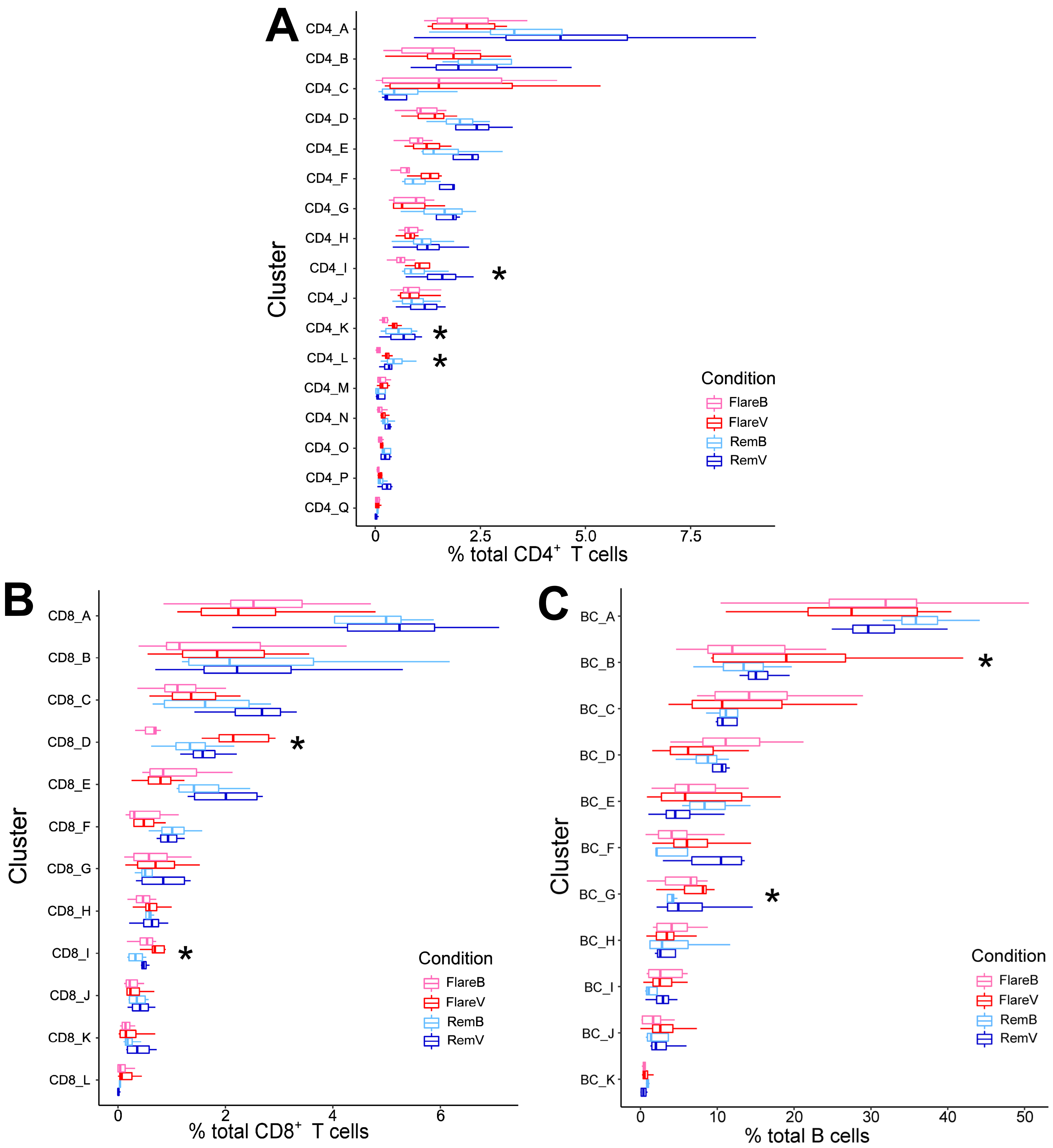

Supplementary Figure 3. Boxplots depicting proportional abundance of circulating lymphocyte clusters within different patient and visit groups. A: $CD4^{+}CD45RO^{+}PD1^{hi}$ T cells clusters. B: $CD8^{+}CD45RO^{+}PD1^{hi}$ T cells clusters. C: B cell clusters. Asterisks indicate statistically significant differences between FlareV and FlareB visits (two-sided $p<0.05$, Wilcoxon rank sum test with Benjamini-Hochberg correction within each cell type). Exact adjusted p values for FlareV vs FlareB contrast as follows: cluster CD4_I: p = 0.044; cluster CD4_K: p = 0.044; cluster CD4_L: p = 0.044; cluster CD8_D: p = 0.047; cluster CD8_I: p = 0.047; cluster BC_B: p = 0.043;cluster BC_G: p = 0.043. FlareB: flare patient, baseline visit; FlareV: flare patient, flare visit; RemB: remission patient, baseline visit; RemV: remission patient, month 6 visit. Box plots represent data from n = 12 patients (8 flare, 4 remission) where the lower bound of lower whisker shows the minimum, lower bound of box shows the lower quartile, centre of box shows the median, upper bound of box shows the upper quartile, and upper bound of upper whisker shows the maximum. Source data are provided as a Source Data file.

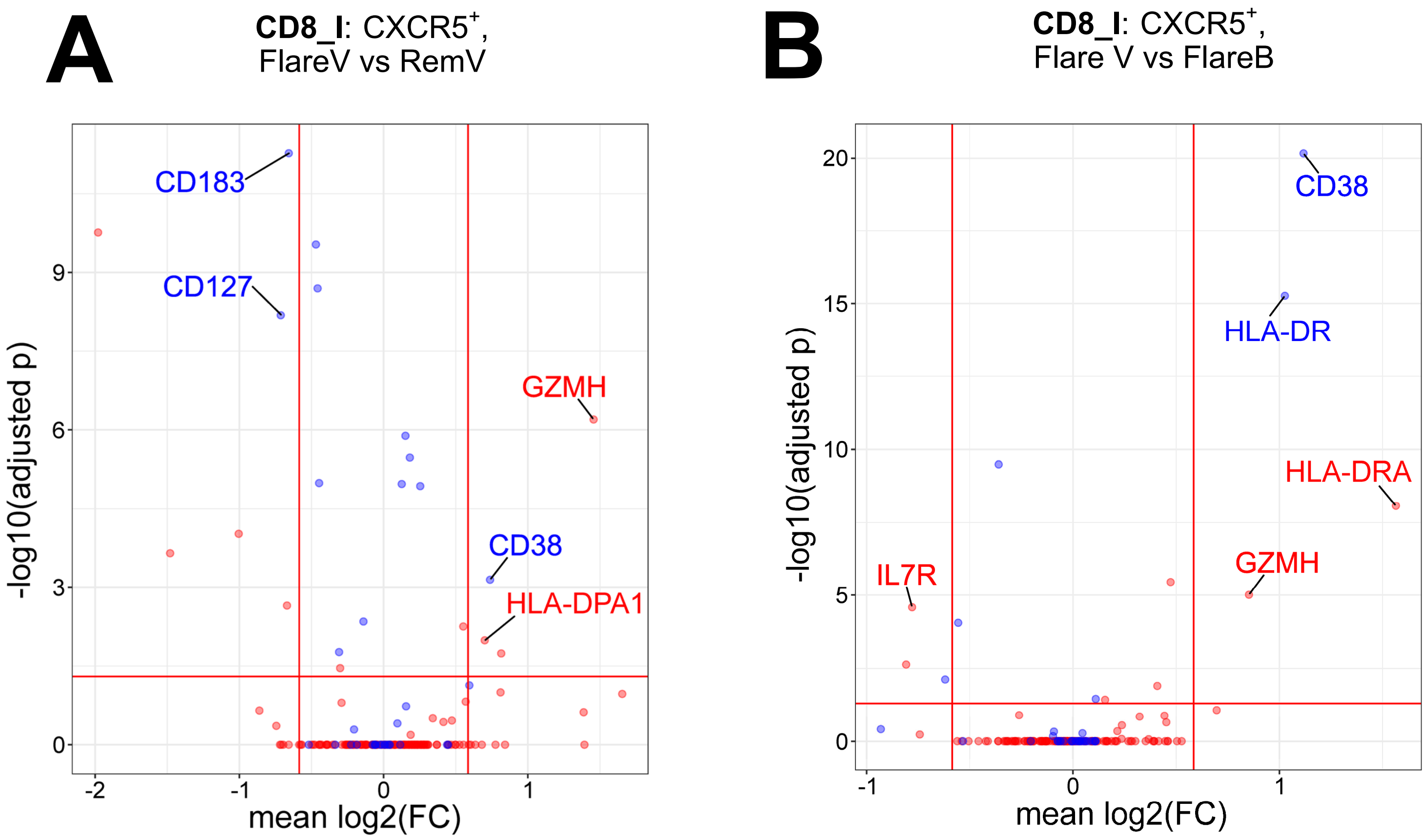

Supplementary Figure 4. Differential gene and surface protein expression within $CD8^+CD45RO^+PD1^{hi}CXCR5^+$ T cells at flare onset. A: FlareV versus RemV visits. B: FlareV versus FlareB visits. The horizontal red line indicates adjusted two-sided p<0.05 threshold (Wilcoxon rank sum test with Bonferroni correction); the vertical lines indicate ±1.5 fold change thresholds. Positive fold change values indicate an increased expression for the first group relative to second group within each contrast. Transcripts are shown in red and surface proteins in blue. Markers of interest as discussed in the results section are highlighted for reference. FlareB: flare patient, baseline visit; FlareV: flare patient, flare visit; RemV: remission patient, month 6 visit. Source data are provided as a Source Data file.

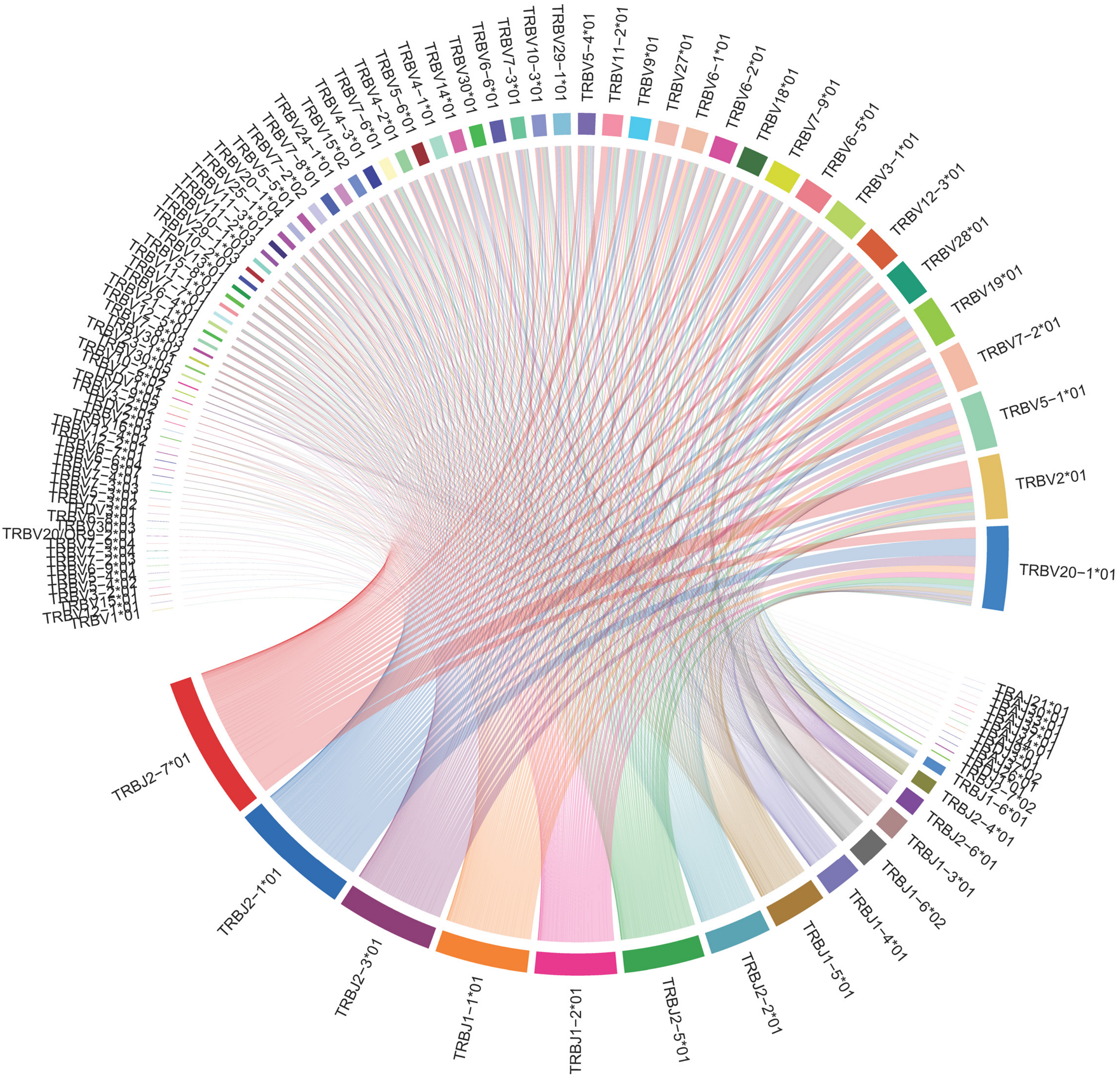

Supplementary Figure 5. V-J gene pairing for alpha/gamma TCRs in CD4^{+}CD45RO^{+}PD1^{hi} T cells across all patients at all time points.

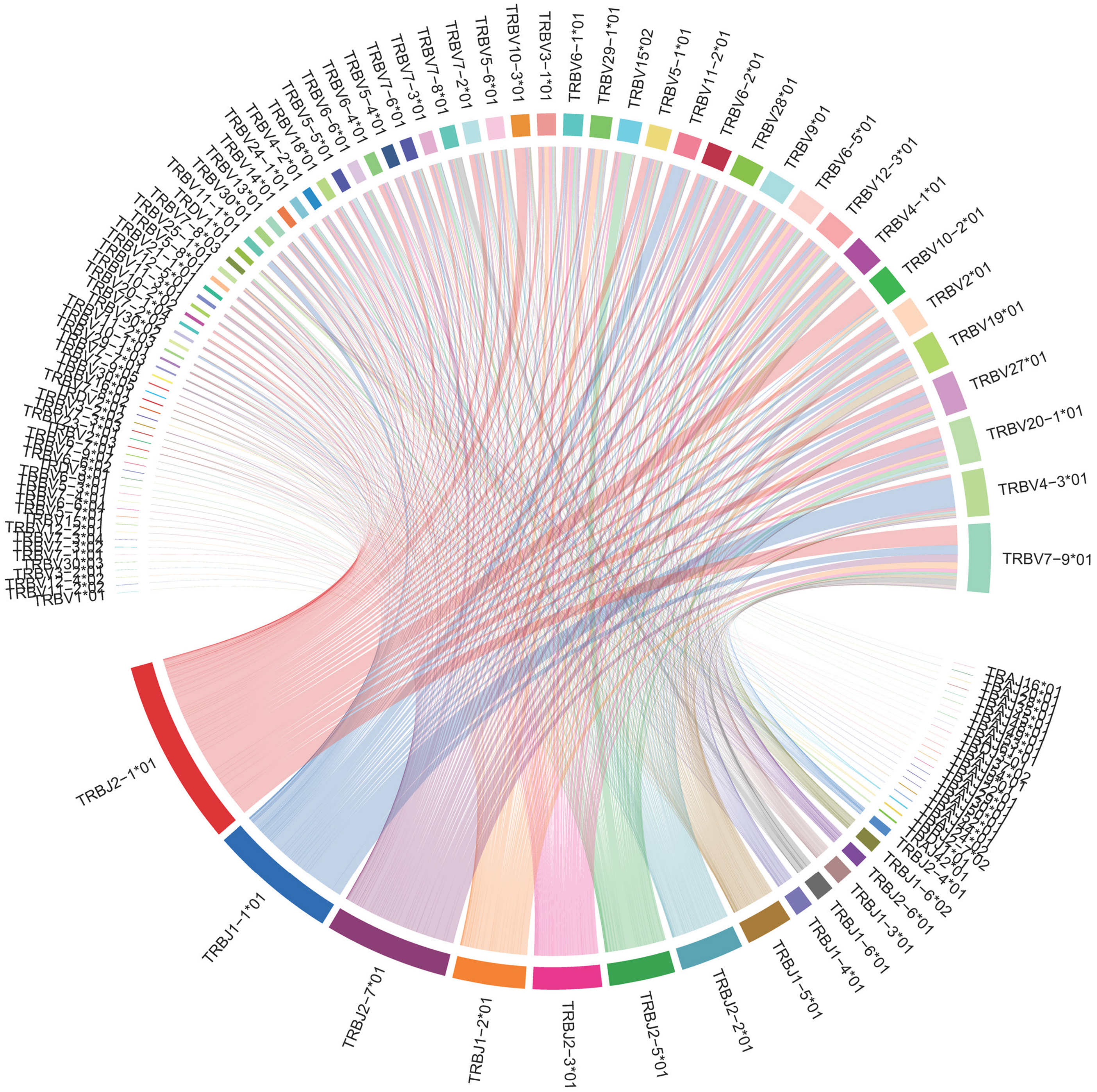

Supplementary Figure 6. V-J gene pairing for alpha/gamma TCRs in CD8^{+}CD45RO^{+}PD1^{hi} T cells across all patients at all time points.

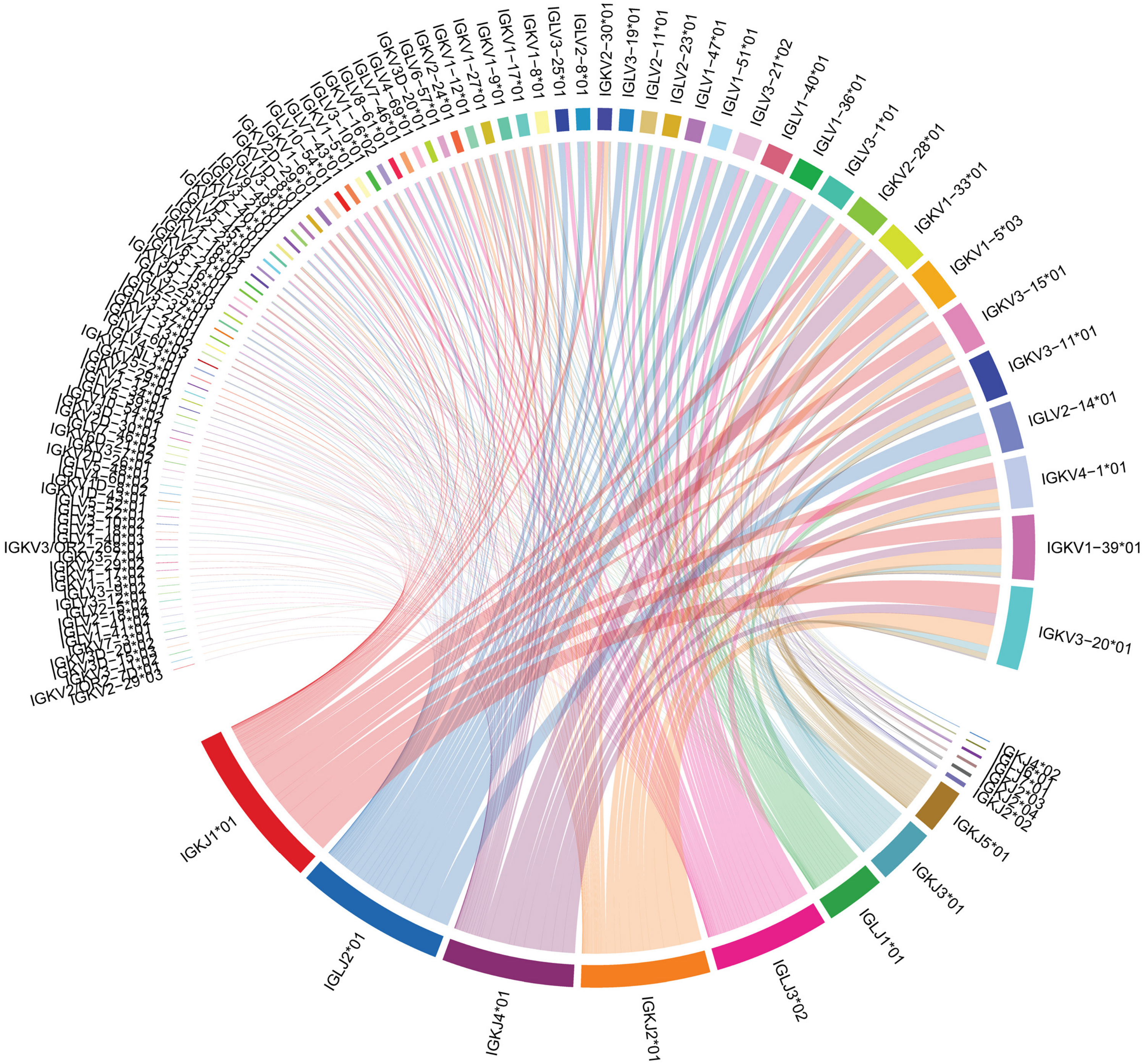

Supplementary Figure 7. Light chain pairing for B cells across all patients at all time points.

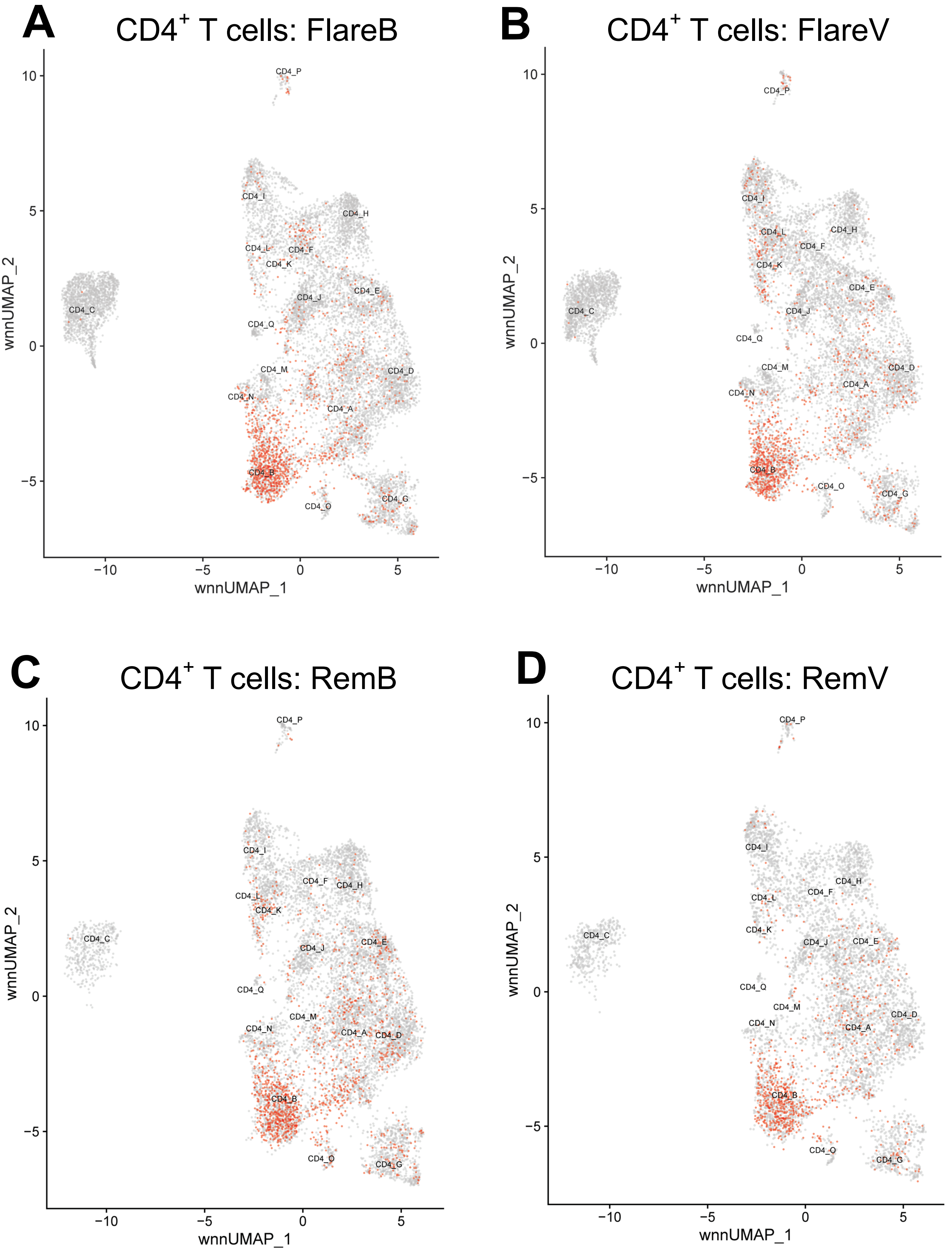

Supplementary Figure 8. UMAPs showing the distribution of clonal CD4^{+}CD45RO^{+}PD1^{hi} T cells by cluster. A: Flare patients at baseline visit (FlareB); B: flare patients at flare visit (FlareV); C: remission patients at baseline visit (RemB); D: remission patients at month 6 visit (RemV). Clonal cells are highlighted in red, and were defined as at least two cells that shared the same paired CDR3 nucleotide sequence within the same patient.

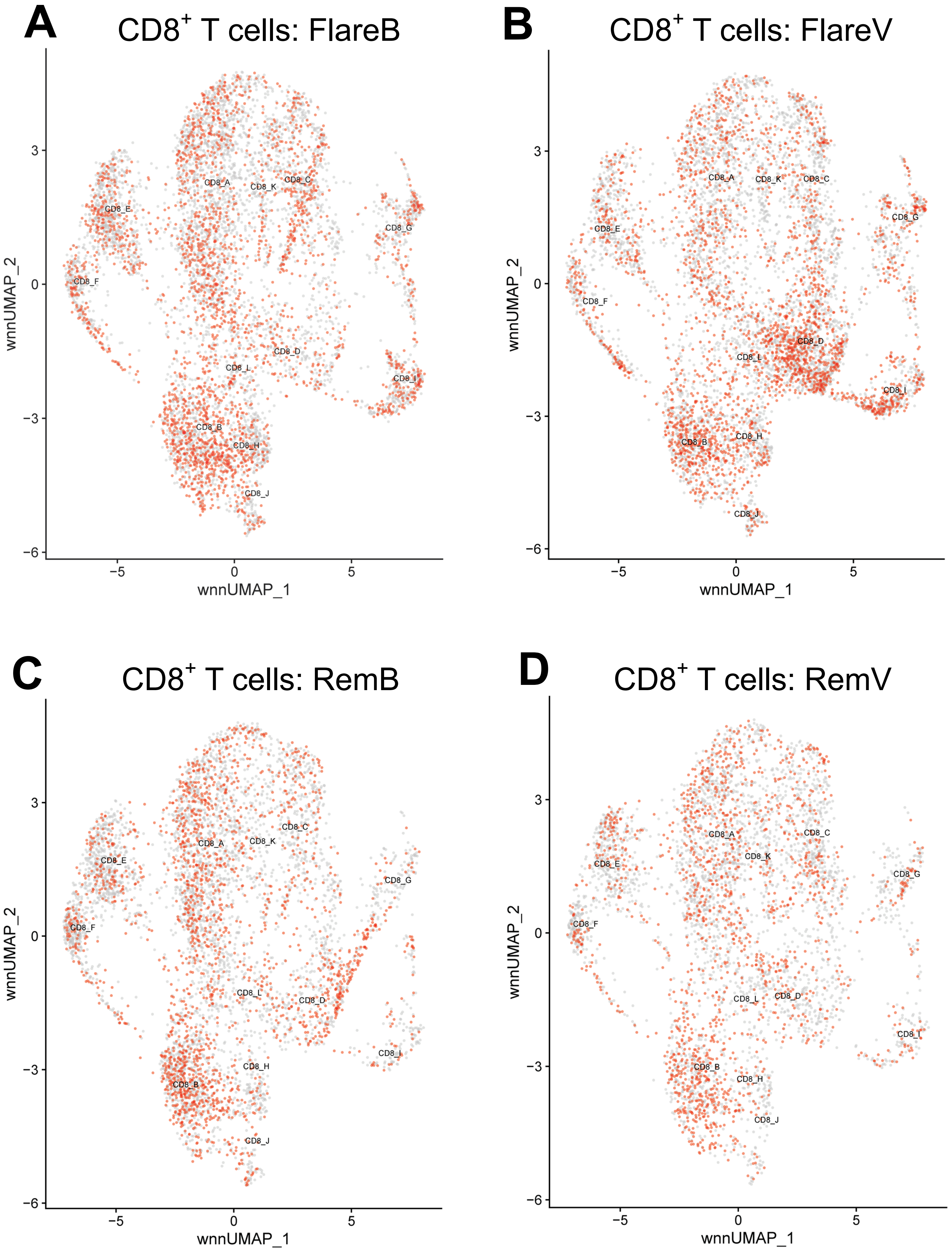

Supplementary Figure 9. UMAPs showing the distribution of clonal CD8$^+$CD45RO$^+$PD1^{hi} T cells by cluster. A: Flare patients at baseline visit (FlareB); B: flare patients at flare visit (FlareV); C: remission patients at baseline visit (RemB); D: remission patients at month 6 visit (RemV). Clonal cells are highlighted in red, and were defined as at least two cells that shared the same paired CDR3 nucleotide sequence within the same patient.

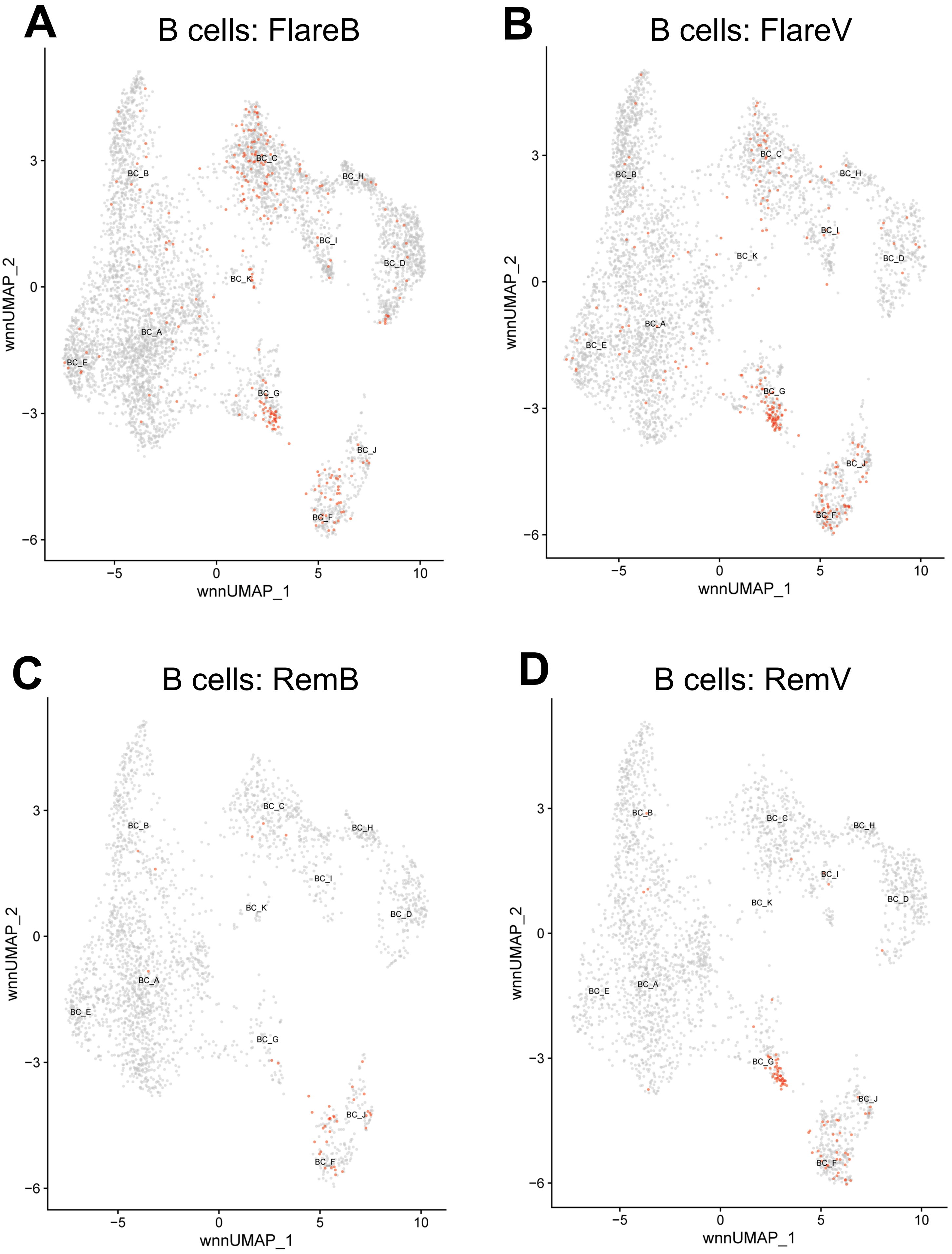

Supplementary Figure 10. UMAPs showing the distribution of clonal B cells by cluster. A: Flare patients at baseline visit (FlareB); B: flare patients at flare visit (FlareV); C: remission patients at baseline visit (RemB); D: remission patients at month 6 visit (RemV). Clonal cells are highlighted in red, and were defined as at least two cells that shared the same paired CDR3 nucleotide sequence within the same patient.

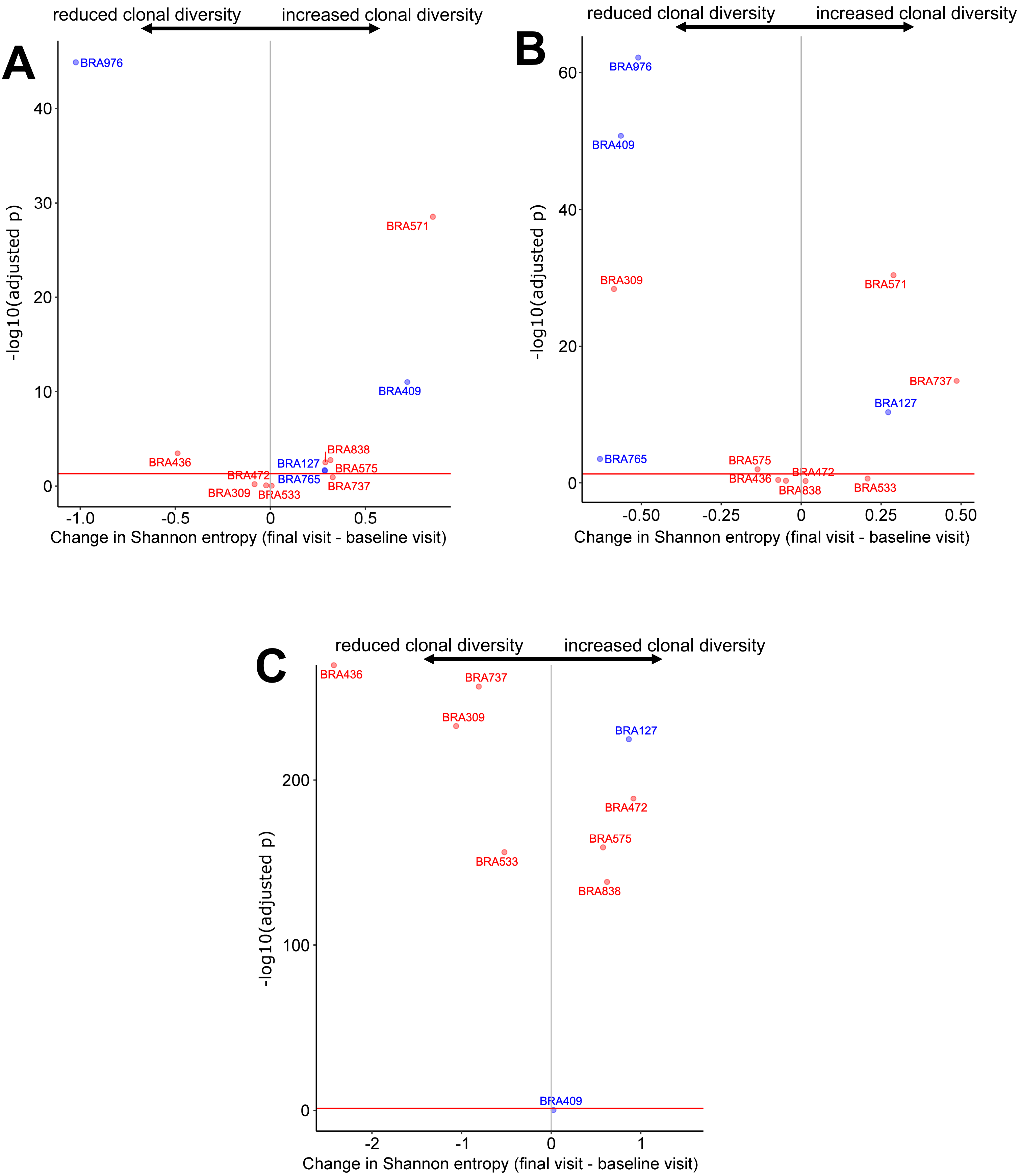

Supplementary Figure 11. Change in clonal diversity (Shannon entropy) between study visits in flare and DFR patients. A: TCR repertoire in $CD8^{+}CD45RO^{+}PD1^{hi}$ T cells. B: TCR repertoire in $CD4^{+}CD45RO^{+}PD1^{hi}$ T cells. C: BCR repertoire in B cells. Each point represents the clonal diversity of all cells from a single patient, with flare patients shown in red and DFR patients shown in blue. Red line shows adjusted two-sided p<0.05 threshold (Hutcheson t test, Benjamini-Hochberg correction). Source data are provided as a Source Data file.

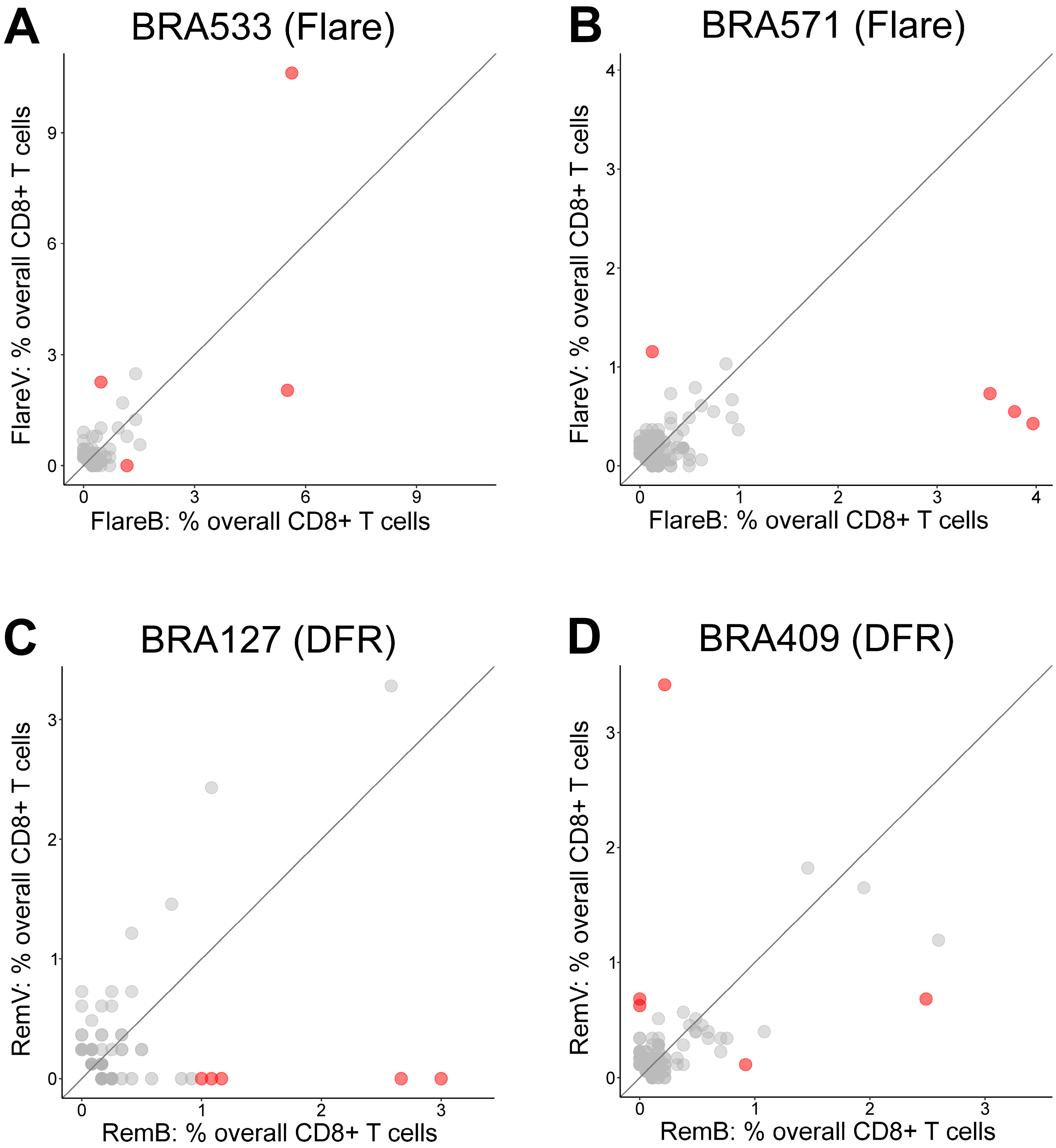

Supplementary Figure 12. Proportional abundance of $CD8^{+}CD45RO^{+}PD1^{hi}$ T cell clones between baseline and final study visits. A: Patient BRA533; B: patient BRA571; C: patient BRA127; D: patient BRA409. Each panel represents an individual patient, with each circle representing an individual clone. Red circles show clones which significantly change in proportional abundance at onset of flare (two-sided $p < 0.05$, Fisher exact test with Benjamini-Hochberg correction). FlareB: flare patient, baseline visit; FlareV: flare patient, flare visit; RemB: remission patient, baseline visit; RemV: remission patient, month 6 visit. Source data are provided as a Source Data file.

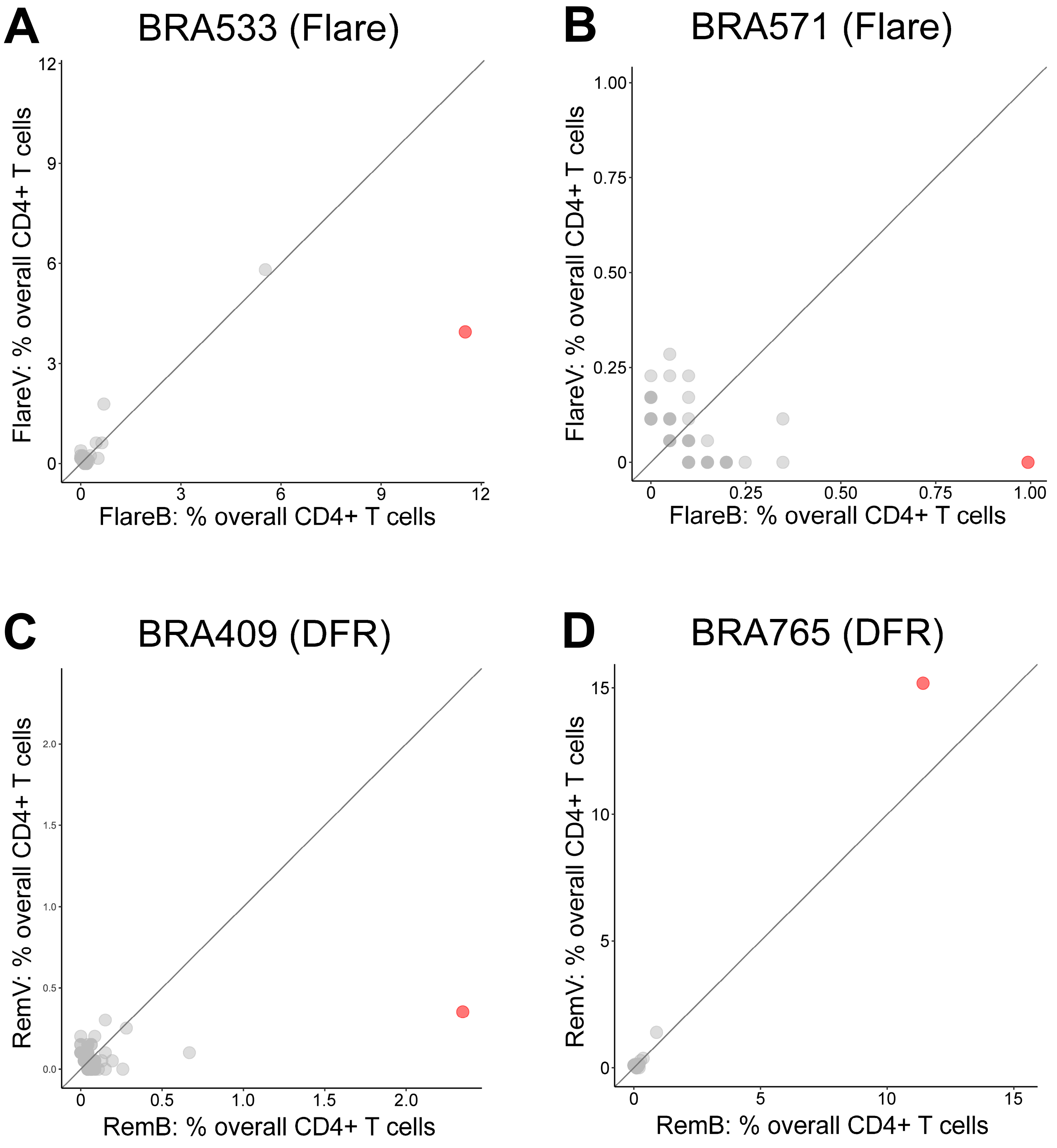

Supplementary Figure 13. Proportional abundance of $CD4^{+}CD45RO^{+}PD1^{hi}$ T cell clones between baseline and final study visits. A: patient BRA533; B: patient BRA571; C: patient BRA409; D: patient BRA765. Each panel represents an individual patient, with each circle representing an individual clone. Red circles show clones which significantly change in proportional abundance at onset of flare (two-sided $p < 0.05$, Fisher exact test with Benjamini-Hochberg correction). FlareB: flare patient, baseline visit; FlareV: flare patient, flare visit; RemB: remission patient, baseline visit; RemV: remission patient, month 6 visit. Source data are provided as a Source Data file.

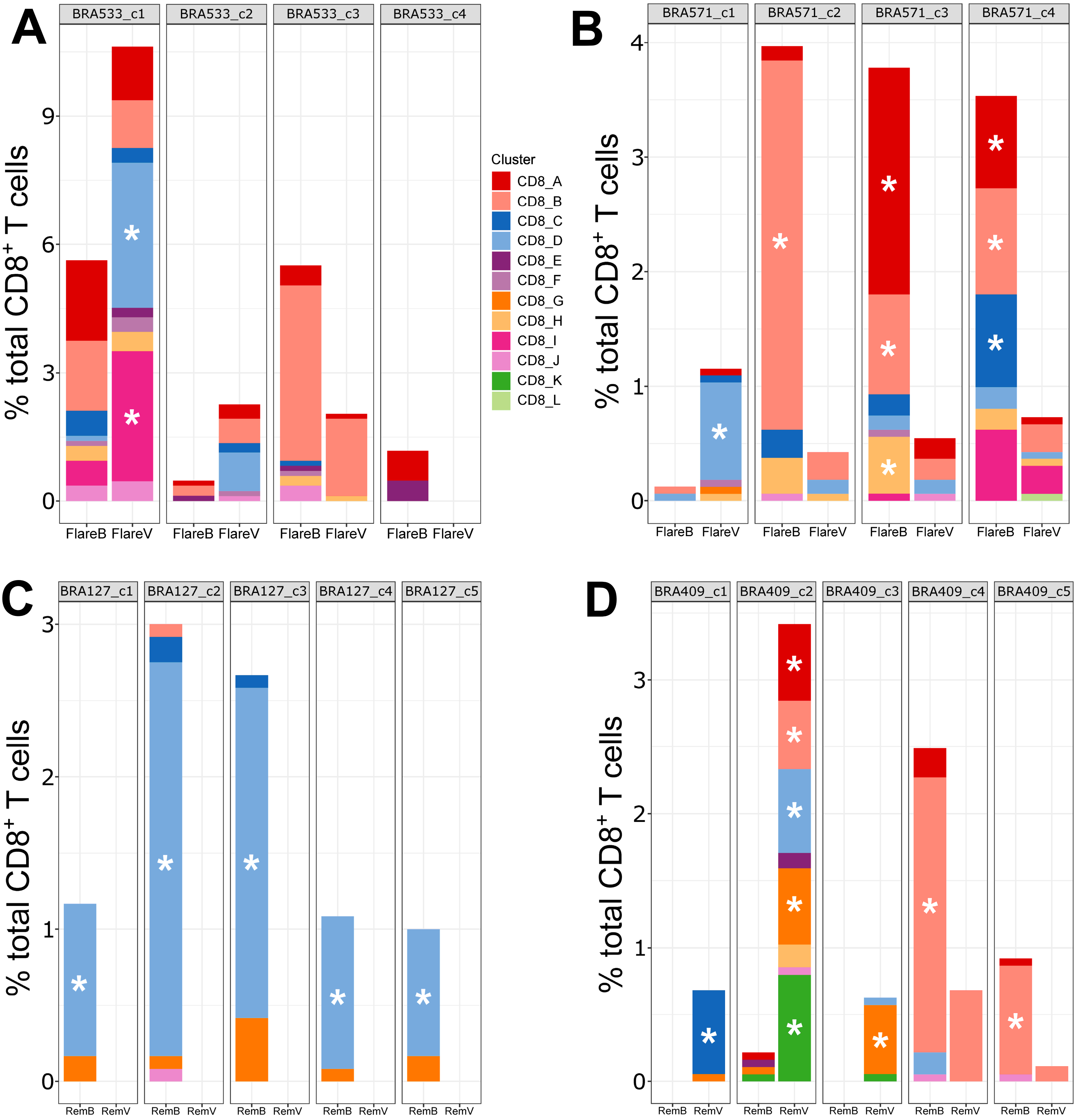

Supplementary Figure 14. Changes in cluster-specific abundances of CD8^{+}CD45RO^{+}PD1^{hi} T cell clones that showed significant longitudinal change in overall proportional abundance after DMARD cessation. A: patient BRA533; B: patient BRA571, C: patient BRA127; D: patient BRA409. Each panel represents an individual clone from an individual patient, asterisks show significant change in cluster-specific proportional abundance at onset of flare (two-sided $p < 0.05$, Fisher exact test with Benjamini-Hochberg correction). FlareB: flare patient, baseline visit; FlareV: flare patient, flare visit; RemB: remission patient, baseline visit; RemV: remission patient, month 6 visit. Source data are provided as a Source Data file.

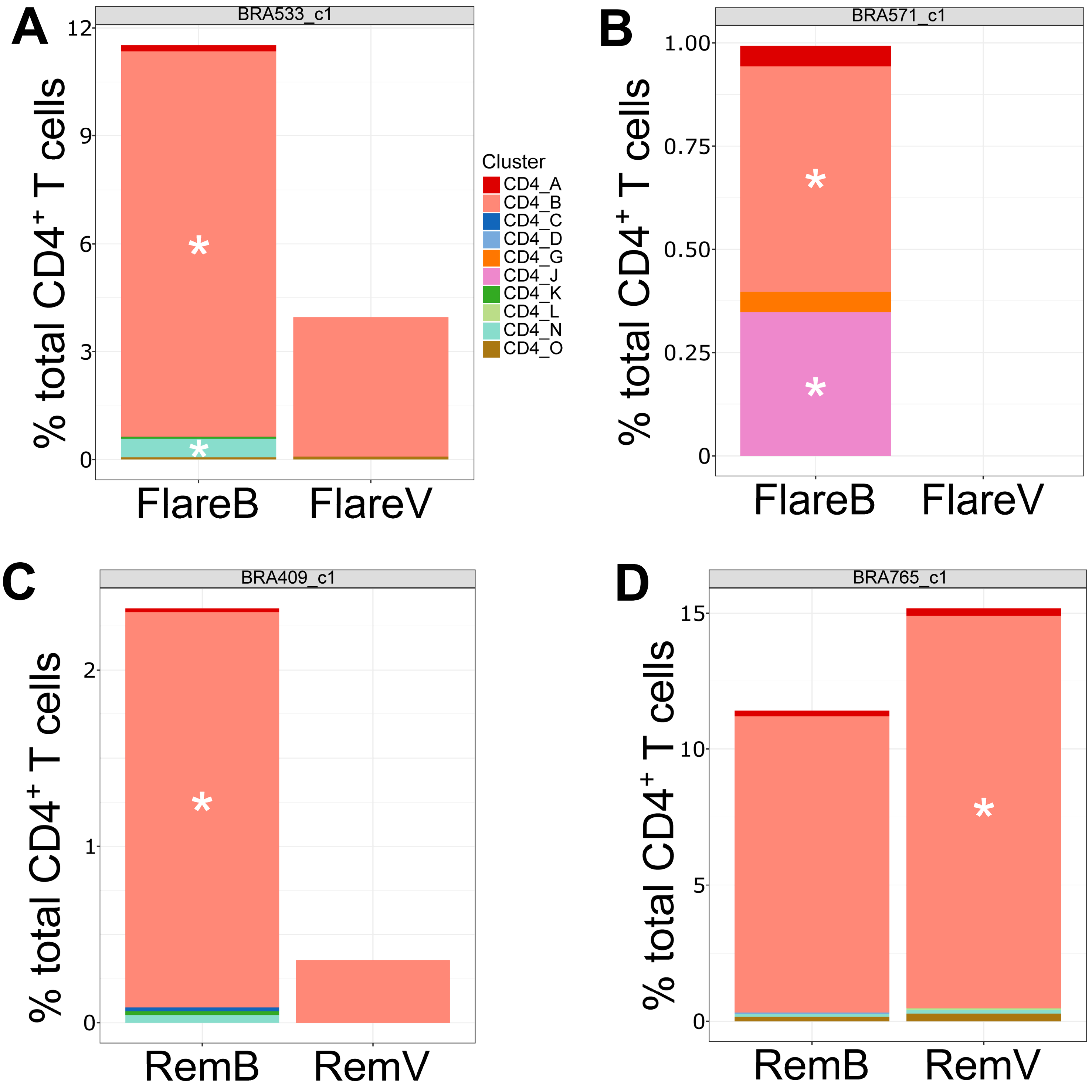

Supplementary Figure 15. Changes in cluster-specific abundances of CD4$^+$CD45RO$^+$PD1^{hi} T cell clones that showed significant longitudinal change in overall proportional abundance after DMARD cessation. A: patient BRA533; B: patient BRA571; C: patient BRA409; D: patient BRA765. Each panel represents an individual clone from an individual patient, asterisks show significant change in cluster-specific proportional abundance at onset of flare (two-sided $p < 0.05$, Fisher exact test with Benjamini-Hochberg correction). FlareB: flare patient, baseline visit; FlareV: flare patient, flare visit; RemB: remission patient, baseline visit; RemV: remission patient, month 6 visit. Source data are provided as a Source Data file.

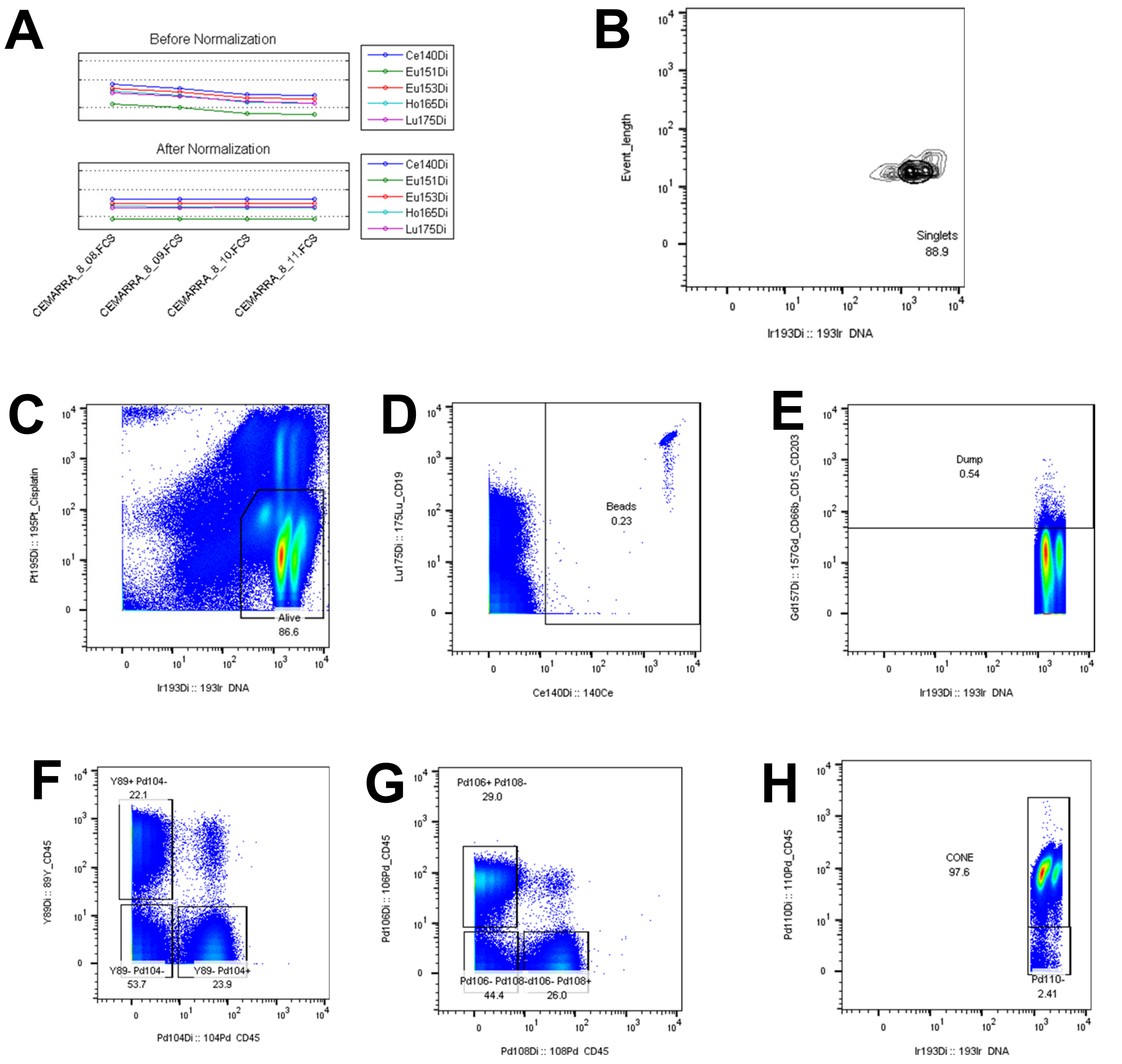

Supplementary Figure 16. Representative images of mass cytometry raw data pre-processing. (**A**) Median compensation bead intensity of four consecutive sample runs before and after normalisation. (**B**) Gating of singlets based on iridium staining and event length. (**C**) Gating of live cells based on iridium and cisplatin staining. (**D**) Removal of Ce140 compensation beads. (**E**) Removal of Gd157 dump channel stain (for CD15$^+$, CD66b$^+$ and CD203$^+$ cells). (**F** to **H**) Sample demultiplexing based on CD45 barcoding of all cells (**F**), Y89$^-$Pd104$^-$ cells (**G**) and Y89$^-$Pd104$^-$Pd106$^-$Pd108$^-$ cells (**H**).

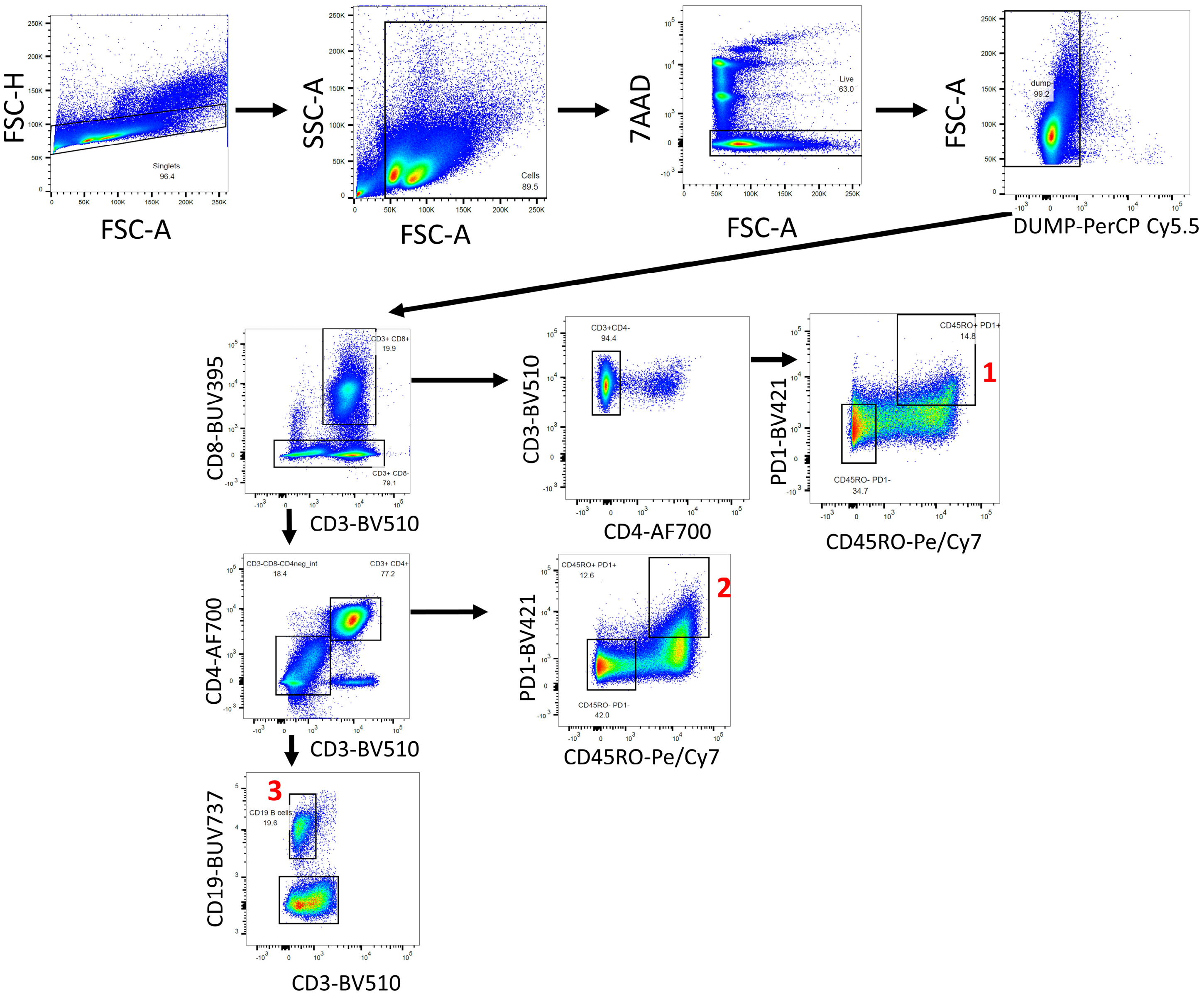

Supplementary Figure 17. Representative images of fluorescence-active cell sorting manual gating strategy. Cells were first gated on forward scatter height (FSC-H) vs forward scatter area (FSC-A) to remove multiplets, followed by side scatter area (SSC-A) vs forward scatter area (FSC-A) gating to remove debris, followed by removal of 7-aminoactinomycin (7AAD) negative dead cells and dump channel (CD66b/CD15/CD203c) positive cells. The resulting cell population was then sequentially gated as shown to isolate $CD3^{+}CD8^{+}CD4^{-}CD45RO^{+}PD1^{hi}$ T cells (population 1), $CD3^{+}CD8^{-}CD4^{+}CD45RO^{+}PD1^{hi}$ T cells (population 2) and $CD19^{+}$ B cells (population 3).

Supplementary Table 1. Number of clones that demonstrated significant longitudinal change in proportional abundance (two-sided adjusted p<0.05, Fisher exact test) at an individual patient level. Dashes (-) indicate no significant changes were observed. Direction of change is described for final sample (FlareV/RemV) versus baseline sample. Individual patient identification numbers are shown in the format BRAXXX. FlareB: flare patient, baseline visit; FlareV: flare patient, flare visit; RemB: remission patient, baseline visit; RemV: remission patient, month 6 visit.

Cell type	Direction	Number of clones with significant change in abundance											
		Flare patients: FlareV vs FlareB								DFR patients: RemV vs RemB			
		BRA309	BRA436	BRA472	BRA533	BRA571	BRA575	BRA737	BRA838	BRA127	BRA409	BRA765	BRA976
$CD4^+$ T cell	Increase	-	-	-	-	-	-	-	-	-	-	1	-
	Decrease	-	-	-	1	1	-	-	-	-	1	-	-
$CD8^+$ T cell	Increase	-	-	-	2	1	-	-	-	-	3	-	-
	Decrease	-	-	-	2	3	-	-	-	5	2	-	-
B cell	Increase	-	-	-	-	-	-	-	-	-	-	-	-
	Decrease	-	-	-	-	-	-	-	-	-	-	-	-

Supplementary Table 2. Mass cytometry antibody panel.

Metal	Marker	Vendor	Catalogue	Clone	Titration
89-Y*	CD45 (barcoding)	Standard Biotools	3089003B	HI30	1:50
104-Pd	CD45 (barcoding)	Biolegend	304002	HI30	1:25
106-Pd	CD45 (barcoding)	Biolegend	304002	HI30	1:25
108-Pd	CD45 (barcoding)	Biolegend	304002	HI30	1:25
110-Pd	CD45 (barcoding)	Biolegend	304002	HI30	1:25
113-In	CD56	Biolegend	318302	HCD56	1:60
115-In	CD8a	Biolegend	301002	RPA-T8	1:60
141-Pr	CD3	Biolegend	300402	UCHT1	1:60
142-Nd	LDLR	BD Biosciences	565641	C7	1:60
143-Nd	CD25	Biolegend	302602	BC96	1:60
144-Nd	IgD	Biolegend	348202	IA6-2	1:60
145-Nd	Ki67	Biolegend	350502	Ki-67	1:60
146-Nd	CD127	Biolegend	351302	A019D5	1:60
147-Sm	CD152 (CTLA4)	Biolegend	369602	BNI3	1:30
148-Nd	CD24	Biolegend	311102	ML5	1:60
149-Sm	CD45RO	Biolegend	304202	UCHL1	1:60
150-Nd	CD11c	Biolegend	301602	3.9	1:60
151-Eu	CD278 (ICOS)	Biolegend	313502	C398.4A	1:60
152-Sm	CD183 (CXCR3)	Biolegend	353702	G025H7	1:60
153-Eu	CD28	Biolegend	302902	CD28.2	1:60
154-Sm	CD370 (CLEC9A)	Biolegend	353802	8F9	1:60
155-Gd	CD45RA	Biolegend	304102	HI100	1:60
156-Gd	CD27	Biolegend	302802	O323	1:60
157-Gd	CD66b	Biolegend	392902	6/40c	1:60
157-Gd	CD15	Biolegend	323002	W6D3	1:60
157-Gd	CD203c	Biolegend	324602	NP4D6	1:60
158-Gd	CD109	BD Biosciences	556039	TEA 2/16	1:60
159-Tb	Foxp3	Biolegend	320102	206D	1:60
160-Gd	CD21	Biolegend	354902	Bu32	1:60
161-Dy	T-bet	BD Biosciences	561263	O4-46	1:60
162-Dy	CD1c	Biolegend	331502	L161	1:60
163-Dy	BATF	Biolegend	654802	9B5A13	1:60
164-Dy	CD14	Biolegend	301802	M5E2	1:60
165-Ho	BCL6	BD Biosciences	561520	K112-91	1:60
166-Er	CD86	Biolegend	305402	IT2.2	1:60
167-Er	GATA3	Biolegend	653802	16E10A23	1:60
168-Er	CD38	Biolegend	303502	HIT2	1:60
169-Tm	RORgT	Miltenyi	130-108-059	REA278	1:60
170-Er	CD123	Biolegend	306002	6H6	1:60
171-Yb	CD279 (PD-1)	Biolegend	329902	EH12.2H7	1:60
172-Yb	HLA-DR	Biolegend	307602	L243	1:60
173-Yb	CD185 (CXCR5)	Biolegend	356902	J252D4	1:60
174-Yb	LAP-TGFbeta	R&D systems	MAB2463	27232	1:60
175-Lu	CD19	Biolegend	302202	HIB19	1:60
176-Yb	CD4	Biolegend	300502	RPA-T4	1:60
193-Ir	DNA-Intercalator				
195-Pt	Viability stain				
209-Bi*	CD16	Standard Biotools	3209002B	3G8	1:120

Metal conjugation performed in-house unless indicated by asterisk (*) where supplied by manufacturer.

Supplementary Table 3. Fluorescence-activated cell sorting antibody panel.

Marker	Fluorochrome	Vendor	Clone	Catalogue	Titration	Laser
CD3	BV510	Biolegend	UCHT1	300448	1:20	405 525/50
CD4	AF700	Biolegend	SK3	344622	1:100	640 730/45
CD8	BUV395	BD Biosciences	RPA-T8	563795	1:20	355 379/28
CD19	BUV737	BD Biosciences	HIB19	741829	1:100	355 730/45
CD45RO	Pe/Cy7	Biolegend	UCHL1	304230	1:20	561 780/60
PD1	BV421	BD Biosciences	MIH4	564323	1:10	405 450/50
CD66b	PerCP Cy5.5	Biolegend	G10F5	305108	1:10	488 710/50
CD15	PerCP Cy5.5	Biolegend	W6D3	323020	1:10	488 710/50
CD203c	PerCP Cy5.5	Biolegend	NP4D6	324608	1:10	488 710/50

Supplementary Table 4. Single-cell RNA sequencing oligo-tagged antibody panel.

Marker	Vendor	Clone	Catalogue
CD278	BD Biosciences	DX29	940043
CD28	BD Biosciences	CD28.2	940017
CD185	BD Biosciences	RF8B2	940042
CD45RA	BD Biosciences	HI100	940011
CD192	BD Biosciences	LS132.1D9	940286
CD195	BD Biosciences	2D7/CCR5	940050
CD25	BD Biosciences	2A3	940009
CD197	BD Biosciences	3D12	940014
CD184	BD Biosciences	12G5	940056
CD127	BD Biosciences	HIL-7R-M21	940012
CD183	BD Biosciences	1C6/CXCR3	940030
CD196	BD Biosciences	11A9	940033
CD223	BD Biosciences	T47-530	940080
CD366	BD Biosciences	7D3	940066
CD62L	BD Biosciences	DREG-56	940041
CD161	BD Biosciences	HP-3G10	940283
CD69	BD Biosciences	FN50	940019
CD274	BD Biosciences	MIH1	940035
CD134	BD Biosciences	ACT35	940060
CD154	BD Biosciences	TRAP1	940053
CD272	BD Biosciences	J168-540	940105
CD103	BD Biosciences	BER-ACT8	940067
CD122	BD Biosciences	MIK-BETA3	940232
CD24	BD Biosciences	ML5	940028
CD27	BD Biosciences	M-T271	940018
CD86	BD Biosciences	2331 (FUN-1)	940025
CD38	BD Biosciences	HIT2	940013
HLA-DR	BD Biosciences	G46-6	940010
IgD	BD Biosciences	IA6-2	940026
IgG	BD Biosciences	G18-145	940027
CD20	BD Biosciences	2H7	940016
CD21	BD Biosciences	B-ly4	940048
CD73	BD Biosciences	AD2	940294
CD39	BD Biosciences	TU66	940073
