# Supplementary material for: Single-cell insights into immune dysregulation in rheumatoid arthritis flare versus drug-free remission
Source: Nat Commun. 2024 Feb 5;15:1063. doi: 10.1038/s41467-024-45213-2 (PMC10844292; doi:10.1038/s41467-024-45213-2)
Supplement: Supplementary file 3 — Description of Additional Supplementary Files [file 41467_2024_45213_MOESM3_ESM.pdf]

Description of Additional Supplementary Files

Supplementary Data 1 – Mass cytometry cluster percentage abundances

Supplementary Data 2 – scRNAseq cluster percentage abundances

Supplementary Data 3 – scRNAseq differential expression data for all markers across all subsets for all paired group contrasts

Supplementary Data 4 – scRNAseq library preparation additional primer sequences

Supplementary Data 5 – scRNAseq custom primer sequences
