# Supplementary material for: Single-cell insights into immune dysregulation in rheumatoid arthritis flare versus drug-free remission
Source: Nat Commun. 2024 Feb 5;15:1063. doi: 10.1038/s41467-024-45213-2 (PMC10844292; doi:10.1038/s41467-024-45213-2)
Supplement: Supplementary file 9 — Reporting Summary [file 41467_2024_45213_MOESM9_ESM.pdf]

nature portfolio

Corresponding author(s): Kenneth Baker

Last updated by author(s): Jan 14, 2024

Reporting Summary

Nature Portfolio wishes to improve the reproducibility of the work that we publish. This form provides structure for consistency and transparency in reporting. For further information on Nature Portfolio policies, see our Editorial Policies and the Editorial Policy Checklist.

Statistics

For all statistical analyses, confirm that the following items are present in the figure legend, table legend, main text, or Methods section.

n/a	Confirmed	
☐	☒	The exact sample size (*n*) for each experimental group/condition, given as a discrete number and unit of measurement
☐	☒	A statement on whether measurements were taken from distinct samples or whether the same sample was measured repeatedly
☐	☒	The statistical test(s) used AND whether they are one- or two-sided<br>*Only common tests should be described solely by name; describe more complex techniques in the Methods section.*
☐	☒	A description of all covariates tested
☐	☒	A description of any assumptions or corrections, such as tests of normality and adjustment for multiple comparisons
☐	☒	A full description of the statistical parameters including central tendency (e.g. means) or other basic estimates (e.g. regression coefficient) AND variation (e.g. standard deviation) or associated estimates of uncertainty (e.g. confidence intervals)
☐	☒	For null hypothesis testing, the test statistic (e.g. *F*, *t*, *r*) with confidence intervals, effect sizes, degrees of freedom and *P* value noted<br>*Give P values as exact values whenever suitable.*
☒	☐	For Bayesian analysis, information on the choice of priors and Markov chain Monte Carlo settings
☒	☐	For hierarchical and complex designs, identification of the appropriate level for tests and full reporting of outcomes
☒	☐	Estimates of effect sizes (e.g. Cohen's *d*, Pearson's *r*), indicating how they were calculated

Our web collection on statistics for biologists contains articles on many of the points above.

Software and code

Policy information about availability of computer code

Data collection	Automated normalisation of raw mass cytometry data was performed using the Normalizer (v0.3) Matlab application of Finck et al (Finck R, et al. Normalization of mass cytometry data with bead standards. Cytometry Part A 83A, 483-494). Manual pre-processing of normalised mass cytometry data was performed in FlowJo (version 10, BD). Automated pre-processing of single cell RNAseq data was performed on the SevenBridges cloud platform using the standard BD Rhapsody pipeline as provided by the manufacturer (BD Single Cell Genomics Bioinformatics Handbook, Doc ID: 54169 Rev. 6.0 dated 10/2018, further information available online at: https://scomix.bd.com/hc/en-us/articles/360023044512-BD-AbSeq-Bioinformatics-Guides)
Data analysis	Further processing and analysis of deconvoluted mass cytometry data was performed in the R statistical environment (v3.4). Normalisation of inter-batch variation was achieved by reference to the technical replicate samples using the Normalizebatch() function of the cydar package (v1.2.1). Further analysis was performed according to the published workflow of Nowicka et al. Unsupervised clustering of normalised data using FlowSOM 61 (version 1.10.0) and ConsensusClusterPlus 62 (v1.42.0) packages was performed, using a 15 x 15 self-organising map followed by reduction to 50 clusters. Manual merging of clusters was performed based upon similarity of canonical lineage marker expression.<br><br>Single cell RNA sequencing feature count matrices were processed in R (v4.1.0) according to the standard Seurat (v4.0) workflow with default parameters unless otherwise specified. The weighted nearest neighbour method was to achieve unsupervised clustering of surface protein and mRNA features simultaneously. The FindClusters function was run with a resolution of 0.4 for CD4+ T cell data, and 0.3 for CD8+ T cell and B cell data. Clusters comprised of fewer than 100 cells were excluded from downstream analyses.<br><br>For mass cytometry data, the statistical significance of differences in circulating proportional abundance of clusters between different sample types was achieved by use of a generalised linear mixed model, accounting for both overdispersion and sample pairing, with adjustment for multiple testing within each pairwise sample type contrast by Benjamini-Hochberg correction. Adjusted two-sided p values < 0.05 were

deemed statistically significant.

For single cell RNA sequencing data, differential feature expression between clusters and paired patient group contrasts was assessed by the Wilcoxon rank sum test, with Bonferroni multiple test correction within each cluster and paired contrast, and a fold-change threshold of > ±1.5. Comparison of proportional subset abundance was performed within each cell type (i.e. of total CD4+ T cells, CD8+ T cells, and B cells) by the Wilcoxon rank sum test, with Benjamini-Hochberg multiple test correction within each paired contrast. Clonal diversity was assessed by Shannon entropy, with significance testing using the Hutcheson t test with Benjamini-Hochberg correction. Differential clone abundance within individual patients between paired visits was assessed by the exact Fisher test with Benjamini-Hochberg correction. A two-sided p <0.05 after multiple test correction was deemed statistically significant.

R analysis scripts are deposited at Zenodo [https://doi.org/10.5281/zenodo.10507330].

For manuscripts utilizing custom algorithms or software that are central to the research but not yet described in published literature, software must be made available to editors and reviewers. We strongly encourage code deposition in a community repository (e.g. GitHub). See the Nature Portfolio guidelines for submitting code & software for further information.

Data

Policy information about availability of data

All manuscripts must include a data availability statement. This statement should provide the following information, where applicable:

- Accession codes, unique identifiers, or web links for publicly available datasets
- A description of any restrictions on data availability
- For clinical datasets or third party data, please ensure that the statement adheres to our policy

The raw and processed single cell RNA sequencing data generated in this study have been deposited at the NCBI Gene Expression Omnibus under accession code GSE245403 [https://www.ncbi.nlm.nih.gov/geo/query/acc.cgi?&acc=GSE245403]. Percentage cluster abundance data from the mass cytometry and single cell RNA sequencing analyses, and differential marker abundance data from the single cell RNA sequencing analysis, have been deposited at Zenodo [https://doi.org/10.5281/zenodo.10507330].

Research involving human participants, their data, or biological material

Policy information about studies with human participants or human data. See also policy information about sex, gender (identity/presentation), and sexual orientation and race, ethnicity and racism.

Reporting on sex and gender	Sex was recorded as documented in the patient's clinical records, and is presented as a demographic in Table 1 of the manuscript. Individual patient level data cannot be presented in this manuscript as would contravene privacy regulations. The small sample size of this cohort precludes sub-analyses by sex.
Reporting on race, ethnicity, or other socially relevant groupings	We do not report on the effects of race, ethnicity or other socially relevant groupings in this manuscript
Population characteristics	Demographic characteristics are presented in Table 1 of the manuscript
Recruitment	Patients were recruited by referral from their rheumatology physician during routine outpatient clinic visits.
Ethics oversight	The BioRRA study protocol was approved by the North East - Tyne & Wear South Research Ethics Committee (National Health Service Health Research Authority, reference 14/NE/1042). The use of BioRRA study samples for the work presented in this manuscript was overseen by the Newcastle Biobank Committee under approval of the North East – Newcastle & North Tyneside 1 Research Ethics Committee (17/NE/0361).

Note that full information on the approval of the study protocol must also be provided in the manuscript.

Field-specific reporting

Please select the one below that is the best fit for your research. If you are not sure, read the appropriate sections before making your selection.

[x] Life sciences [ ] Behavioural & social sciences [ ] Ecological, evolutionary & environmental sciences

For a reference copy of the document with all sections, see nature.com/documents/nr-reporting-summary-flat.pdf

Life sciences study design

All studies must disclose on these points even when the disclosure is negative.

Sample size	This manuscript details exploratory analyses of data from the Biomarkers of Remission in Rheumatoid Arthritis (BioRRA) study, and as such this study was not powered a priori for these analyses. Sample size was thus determined by the available cryopreserved samples remaining from the study.
Data exclusions	No data was excluded from our analyses.
Replication	The raw data and R code used in our analyses is available for replication purposes.

Randomization	The BioRRA study was a non-randomised clinical trial, and it was thus not possible to randomise allocation of samples to experimental groups in this study.
Blinding	The BioRRA study was a non-blinded clinical trial, and it was thus not possible to blind the investigators to clinical outcome during data collection and analysis in this study.

Reporting for specific materials, systems and methods

We require information from authors about some types of materials, experimental systems and methods used in many studies. Here, indicate whether each material, system or method listed is relevant to your study. If you are not sure if a list item applies to your research, read the appropriate section before selecting a response.

Materials & experimental systems

n/a	Involved in the study	
☐	☒	Antibodies
☒	☐	Eukaryotic cell lines
☒	☐	Palaeontology and archaeology
☒	☐	Animals and other organisms
☐	☒	Clinical data
☒	☐	Dual use research of concern
☒	☐	Plants

Methods

n/a	Involved in the study	
☒	☐	ChIP-seq
☐	☒	Flow cytometry
☒	☐	MRI-based neuroimaging

Antibodies

Antibodies used

The antibodies used in the mass cytometry panel are detailed below:
Marker, Vendor, Catalogue number, Clone
CD45,Standard Biotools,3089003B,HI30
CD45,Biolegend,304002,HI30
CD56,Biolegend,318302,HCD56
CD8a,Biolegend,301002,RPA-T8
CD3,Biolegend,300402,UCHT1
LDLR,BD,Bioscience,565641,C7
CD25,Biolegend,302602,BC96
IgD,Biolegend,348202,IA6-2
Ki67,Biolegend,350502,Ki-67
CD127,Biolegend,351302,A019D5
CD152 (CTLA4),Biolegend,369602,BNI3
CD24,Biolegend,311102,ML5
CD45RO,Biolegend,304202,UCHL1
CD11c,Biolegend,301602,3.9
CD278 (ICOS),Biolegend,313502,C398.4A
CD183 (CXCR3),Biolegend,353702,G025H7
CD28,Biolegend,302902,CD28.2
CD370 (CLEC9A),Biolegend,353802,8F9
CD45RA,Biolegend,304102,HI100
CD27,Biolegend,302802,O323
CD66b,Biolegend,392902,6/40c
CD15,Biolegend,323002,W6D3
CD203c,Biolegend,324602,NP4D6
CD109,BD,Bioscience,556039,TEA,2/16
Foxp3,Biolegend,320102,206D
CD21,Biolegend,354902,Bu32
T-bet,BD,Bioscience,561263,O4-46
CD1c,Biolegend,331502,L161
BATF,Biolegend,654802,9B5A13
CD14,Biolegend,301802,M5E2
BCL6,BD,Biosciences,561520,K112-91
CD86,Biolegend,305402,IT2.2
GATA3,Biolegend,653802,16E10A23
CD38,Biolegend,303502,HIT2
RORgT,Miltenyi,130-108-059,REA278
CD123,Biolegend,306002,6H6
CD279 (PD-1),Biolegend,329902,EH12.2H7
HLA-DR,Biolegend,307602,L243

CD185 (CXCR5),Biolegend,356902,J252D4
LAP-TGFbeta,R&D systems,MAB2463,27232
CD19,Biolegend,302202,HIB19
CD4,Biolegend,300502,RPA-T4
CD16,Fluidigm,3209002B,3G8

The antibodies used in the FACS panel are detailed below.
Marker,Fluorochrome,Vendor,Clone,Catalogue number
CD3,BV510,Biolegend,UCHT1,300448
CD4,AF700,Biolegend,SK3,344622
CD8,BUV395,BD,RPA-T8,563795
CD19,BUV737,BD,HIB19,741829
CD45RO,Pe/Cy7,Biolegend,UCHL1,304230
PD1,BV421,BD,MIH4,564323
CD66b,PerCP Cy5.5,Biolegend,G10F5,305108
CD15,PerCP Cy5.5,Biolegend,W6D3,323020
CD203c,PerCP Cy5.5,Biolegend,NP4D6,324608

The antibodies used in the single cell RNA sequencing oligo-tagged antibody panel are detailed below.
Marker,Vendor,Clone,Catalogue number
CD278,BD,DX29,940043
CD28,BD,CD28.2,940017
CD185,BD,RF8B2,940042
CD45RA,BD,HI100,940011
CD192,BD,LS132.1D9,940286
CD195,BD,2D7/CCR5,940050
CD25,BD,2A3,940009
CD197,BD,3D12,940014
CD184,BD,12G5,940056
CD127,BD,HIL-7R-M21,940012
CD183,BD,1C6/CXCR3,940030
CD196,BD,11A9,940033
CD223,BD,T47-530,940080
CD366,BD,7D3,940066
CD62L,BD,DREG-56,940041
CD161,BD,HP-3G10,940283
CD69,BD,FN50,940019
CD274,BD,MIH1,940035
CD134,BD,ACT35,940060
CD154,BD,TRAP1,940053
CD272,BD,J168-540,940105
CD103,BD,BER-ACT8,940067
CD122,BD,MIK-BETA3,940232
CD24,BD,ML5,940028
CD27,BD,M-T271,940018
CD86,BD,2331 (FUN-1),940025
CD38,BD,HIT2,940013
HLA-DR,BD,G46-6,940010
IgD,BD,IA6-2,940026
IgG,BD,G18-145,940027
CD20,BD,2H7,940016
CD21,BD,B-ly4,940048
CD73,BD,AD2,940294
CD39,BD,TU66,940073

Validation

All antibodies were validated for their respective target protein reactivity in human samples by their respective manufacturers. URLs for the manufacturer's websites which include antibody data sheets are provided below.
Mass cytometry panel:
CD45: https://store.standardbio.com/Cytometry/ConsumablesandReagentsCytometry/MaxparAntibodies/Anti-Human%20CD45%20-HI30-89Y%E2%80%94100%20Tests
CD45: https://www.biolegend.com/en-gb/cell-separation/purified-anti-human-cd45-antibody-710
CD56: https://www.biolegend.com/en-gb/products/purified-anti-human-cd56-ncam-antibody-3794
CD8a: https://www.biolegend.com/en-gb/products/purified-anti-human-cd8a-antibody-839
CD3: https://www.biolegend.com/en-gb/products/purified-anti-human-cd3-antibody-867
LDLR: https://www.bdbiosciences.com/en-gb/products/reagents/flow-cytometry-reagents/research-reagents/single-color-antibodies-ruo/purified-mouse-anti-human-ldlr.565641
CD25: https://www.biolegend.com/en-gb/products/purified-anti-human-cd25-antibody-618
IgD: https://www.biolegend.com/en-gb/products/purified-anti-human-igd-antibody-6527
Ki67: https://www.biolegend.com/en-gb/products/purified-anti-human-ki-67-antibody-6967

CD127: https://www.biolegend.com/en-gb/products/purified-anti-human-cd127-il-7ralpha-antibody-7093
CD152 (CTLA4): https://www.biolegend.com/en-gb/products/purified-anti-human-cd152-ctla-4-antibody-12627
CD24: https://www.biolegend.com/en-gb/products/purified-anti-human-cd24-antibody-1806
CD45RO: https://www.biolegend.com/en-gb/products/purified-anti-human-cd45ro-antibody-860
CD11c: https://www.biolegend.com/en-gb/products/purified-anti-human-cd11c-antibody-564
CD278 (ICOS): https://www.biolegend.com/en-gb/products/purified-anti-human-mouse-rat-cd278-icos-antibody-2477
CD183 (CXCR3): https://www.biolegend.com/en-gb/products/purified-anti-human-cd183-cxcr3-antibody-7577
CD28: https://www.biolegend.com/en-gb/products/purified-anti-human-cd28-antibody-632
CD370 (CLEC9A): https://www.biolegend.com/en-gb/products/purified-anti-human-cd370-clec9a-dngr1-antibody-7600
CD45RA: https://www.biolegend.com/en-gb/products/purified-anti-human-cd45ra-antibody-689
CD27: https://www.biolegend.com/en-gb/products/purified-anti-human-cd27-antibody-812
CD66b: https://www.biolegend.com/en-gb/products/purified-anti-human-cd66b-antibody-15433
CD15: https://www.biolegend.com/en-gb/products/purified-anti-human-cd15-ssea-1-antibody-3699
CD203c: https://www.biolegend.com/en-gb/products/purified-anti-human-cd203c-e-npp3-antibody-3737
CD109: https://www.bdbiosciences.com/en-gb/products/reagents/flow-cytometry-reagents/research-reagents/single-color-antibodies-ruo/purified-mouse-anti-human-cd109.556039
Foxp3: https://www.biolegend.com/en-gb/products/purified-anti-human-foxp3-antibody-2897
CD21: https://www.biolegend.com/en-gb/products/purified-anti-human-cd21-antibody-8032
T-bet: https://www.bdbiosciences.com/en-gb/products/reagents/microscopy-imaging-reagents/immunohistochemistry-reagents/purified-mouse-anti-t-bet.561263
CD1c: https://www.biolegend.com/en-gb/products/purified-anti-human-cd1c-antibody-4836
BATF: https://www.biolegend.com/en-gb/products/purified-anti-batf-antibody-8414
CD14: https://www.biolegend.com/en-gb/products/purified-anti-human-cd14-antibody-797
BCL6: https://www.bdbiosciences.com/en-gb/products/reagents/flow-cytometry-reagents/research-reagents/single-color-antibodies-ruo/purified-mouse-anti-bcl-6.561520
CD86: https://www.biolegend.com/en-gb/products/purified-anti-human-cd86-antibody-780
GATA3: https://www.biolegend.com/en-gb/products/purified-anti-gata3-antibody-8340
CD38: https://www.biolegend.com/en-gb/products/purified-anti-human-cd38-antibody-748
RORgT: https://www.miltenyibiotec.com/GB-en/products/rorg-t-antibody-anti-human-mouse-reafinity-rea278.html#conjugate=vio-b515:size=100-tests-in-200-ul
CD123: https://www.biolegend.com/en-gb/products/purified-anti-human-cd123-antibody-578
CD279 (PD-1): https://www.biolegend.com/en-gb/products/purified-anti-human-cd279-pd-1-antibody-4410
HLA-DR: https://www.biolegend.com/en-gb/products/purified-anti-human-hla-dr-antibody-792
CD185 (CXCR5): https://www.biolegend.com/en-gb/products/purified-anti-human-cd185-cxcr5-antibody-8357
LAP-TGFbeta: https://www.rndsystems.com/products/human-lap-tgf-beta1-antibody-27232_mab2463
CD19: https://www.biolegend.com/en-gb/products/purified-anti-human-cd19-antibody-721
CD4: https://www.biolegend.com/en-gb/products/purified-anti-human-cd4-antibody-830
CD16: https://store.standardbio.com/Cytometry/ConsumablesandReagentsCytometry/MaxparAntibodies/Anti-Human%20CD16%20-3G8-209Bi%E2%80%94100%20Tests

FACS panel:
CD3: https://www.biolegend.com/en-gb/products/brilliant-violet-510-anti-human-cd3-antibody-9792
CD4: https://www.biolegend.com/en-gb/products/alexa-fluor-700-anti-human-cd4-antibody-9354
CD8: https://www.bdbiosciences.com/en-gb/products/reagents/flow-cytometry-reagents/research-reagents/single-color-antibodies-ruo/buv395-mouse-anti-human-cd8.563795
CD19: https://www.bdbiosciences.com/en-gb/products/reagents/flow-cytometry-reagents/research-reagents/single-color-antibodies-ruo/buv737-mouse-anti-human-cd19.741829
CD45RO: https://www.biolegend.com/en-gb/products/pe-cyanine7-anti-human-cd45ro-antibody-7760
PD1: https://www.bdbiosciences.com/en-gb/products/reagents/flow-cytometry-reagents/research-reagents/single-color-antibodies-ruo/bv421-mouse-anti-human-cd279-pd-1.564323
CD66b: https://www.biolegend.com/en-gb/products/percp-cyanine5-5-anti-human-cd66b-antibody-6585
CD15: https://www.biolegend.com/en-gb/products/percp-cyanine5-5-anti-human-cd15-ssea-1-antibody-4249
CD203c: https://www.biolegend.com/en-gb/products/percp-cyanine5-5-anti-human-cd203c-e-npp3-antibody-6443

Single cell RNA sequencing oligo-tagged antibody panel:
CD278: https://www.bdbiosciences.com/en-gb/products/reagents/single-cell-multiomics-reagents/bd-abseq-assay/oligo-mouse-anti-human-cd278-icos.940043
CD28: https://www.bdbiosciences.com/en-gb/products/reagents/single-cell-multiomics-reagents/bd-abseq-assay/oligo-mouse-anti-human-cd28.940017
CD185: https://www.bdbiosciences.com/en-gb/products/reagents/single-cell-multiomics-reagents/bd-abseq-assay/oligo-rat-anti-human-cxcr5-cd185.940042
CD45RA: https://www.bdbiosciences.com/en-gb/products/reagents/single-cell-multiomics-reagents/bd-abseq-assay/oligo-mouse-anti-human-cd45ra.940011
CD192: https://www.bdbiosciences.com/en-gb/products/reagents/single-cell-multiomics-reagents/bd-abseq-assay/oligo-mouse-anti-human-ccr2-cd192.940286
CD195: https://www.bdbiosciences.com/en-gb/products/reagents/single-cell-multiomics-reagents/bd-abseq-assay/oligo-mouse-anti-human-cd195.940050
CD25: https://www.bdbiosciences.com/en-gb/products/reagents/single-cell-multiomics-reagents/bd-abseq-assay/oligo-mouse-anti-human-cd25-il-2-receptor.940009
CD197: https://www.bdbiosciences.com/en-gb/products/reagents/single-cell-multiomics-reagents/bd-abseq-assay/oligo-rat-anti-

human-ccr7-cd197.940014
CD184: https://www.bdbiosciences.com/en-gb/products/reagents/single-cell-multiomics-reagents/bd-abseq-assay/oligo-mouse-anti-human-cd184-cxcr4.940056
CD127: https://www.bdbiosciences.com/en-gb/products/reagents/single-cell-multiomics-reagents/bd-abseq-assay/oligo-mouse-anti-human-cd127.940012
CD183: https://www.bdbiosciences.com/en-gb/products/reagents/single-cell-multiomics-reagents/bd-abseq-assay/oligo-mouse-anti-human-cd183-cxcr3.940030
CD196: https://www.bdbiosciences.com/en-gb/products/reagents/single-cell-multiomics-reagents/bd-abseq-assay/oligo-mouse-anti-human-cd196-ccr6.940033
CD223: https://www.bdbiosciences.com/en-gb/products/reagents/single-cell-multiomics-reagents/bd-abseq-assay/oligo-mouse-anti-human-lag-3-cd223.940080
CD366: https://www.bdbiosciences.com/en-gb/products/reagents/single-cell-multiomics-reagents/bd-abseq-assay/oligo-mouse-anti-human-tim-3-cd366.940066
CD62L: https://www.bdbiosciences.com/en-gb/products/reagents/single-cell-multiomics-reagents/bd-abseq-assay/oligo-mouse-anti-human-cd62l.940041
CD161: https://www.bdbiosciences.com/en-gb/products/reagents/single-cell-multiomics-reagents/bd-abseq-assay/oligo-mouse-anti-human-cd161.940283
CD69: https://www.bdbiosciences.com/en-gb/products/reagents/single-cell-multiomics-reagents/bd-abseq-assay/oligo-mouse-anti-human-cd69.940019
CD274: https://www.bdbiosciences.com/en-gb/products/reagents/single-cell-multiomics-reagents/bd-abseq-assay/oligo-mouse-anti-human-cd274-b7-h1.940035
CD134: https://www.bdbiosciences.com/en-gb/products/reagents/single-cell-multiomics-reagents/bd-abseq-assay/oligo-mouse-anti-human-cd134.940060
CD154: https://www.bdbiosciences.com/en-gb/products/reagents/single-cell-multiomics-reagents/bd-abseq-assay/oligo-mouse-anti-human-cd154.940053
CD272: https://www.bdbiosciences.com/en-gb/products/reagents/single-cell-multiomics-reagents/bd-abseq-assay/oligo-mouse-anti-human-cd272-btla.940105
CD103: https://www.bdbiosciences.com/en-gb/products/reagents/single-cell-multiomics-reagents/bd-abseq-assay/oligo-mouse-anti-human-cd103.940067
CD122: https://www.bdbiosciences.com/en-gb/products/reagents/single-cell-multiomics-reagents/bd-abseq-assay/oligo-mouse-anti-human-cd122.940232
CD24: https://www.bdbiosciences.com/en-gb/products/reagents/single-cell-multiomics-reagents/bd-abseq-assay/oligo-mouse-anti-human-cd24.940028
CD27: https://www.bdbiosciences.com/en-gb/products/reagents/single-cell-multiomics-reagents/bd-abseq-assay/oligo-mouse-anti-human-cd27.940018
CD86: https://www.bdbiosciences.com/en-gb/products/reagents/single-cell-multiomics-reagents/bd-abseq-assay/oligo-mouse-anti-human-cd86-b7-2.940025
CD38: https://www.bdbiosciences.com/en-gb/products/reagents/single-cell-multiomics-reagents/bd-abseq-assay/oligo-mouse-anti-human-cd38.940013
HLA-DR: https://www.bdbiosciences.com/en-gb/products/reagents/single-cell-multiomics-reagents/bd-abseq-assay/oligo-mouse-anti-human-hla-dr.940010
IgD: https://www.bdbiosciences.com/en-gb/products/reagents/single-cell-multiomics-reagents/bd-abseq-assay/oligo-mouse-anti-human-igd.940026
IgG: https://www.bdbiosciences.com/en-gb/products/reagents/single-cell-multiomics-reagents/bd-abseq-assay/oligo-mouse-anti-human-igg.940027
CD20: https://www.bdbiosciences.com/en-gb/products/reagents/single-cell-multiomics-reagents/bd-abseq-assay/oligo-mouse-anti-human-cd20.940016
CD21: https://www.bdbiosciences.com/en-gb/products/reagents/single-cell-multiomics-reagents/bd-abseq-assay/oligo-mouse-anti-human-cd21.940048
CD73: https://www.bdbiosciences.com/en-gb/products/reagents/single-cell-multiomics-reagents/bd-abseq-assay/oligo-mouse-anti-human-cd73.940294
CD39: https://www.bdbiosciences.com/en-gb/products/reagents/single-cell-multiomics-reagents/bd-abseq-assay/oligo-mouse-anti-human-cd39.940073

Clinical data

Policy information about clinical studies
All manuscripts should comply with the ICMJEguidelines for publication of clinical research and a completedCONSORT checklist must be included with all submissions.

Clinical trial registration

The BioRRA study is registered at ClinicalTrials.gov (NCT02219347).

Study protocol

The design of the BioRRA study is described in the original study publication (Baker KF, et al. Predicting drug-free remission in rheumatoid arthritis: A prospective interventional cohort study. J Autoimmun 105, 102298). The original protocol of the BioRRA study is available from the corresponding author upon request.

Data collection

Patients were recruited to the BioRRA study between September 2014 and October 2016. All clinical study procedures were performed at a single research site (Newcastle upon Tyne Hospitals NHS Foundation Trust).

Outcomes

The primary outcome measure of the BioRRA study was time to arthritis flare. Arthritis flare was defined as any single measure of disease activity score in 28 joints with C-reactive protein (DAS28-CRP) ≥ 2.4 during the six month follow-up period after cessation of disease-modifying anti-rheumatic drugs.

Flow Cytometry

Plots

Confirm that:

- [x] The axis labels state the marker and fluorochrome used (e.g. CD4-FITC).
- [x] The axis scales are clearly visible. Include numbers along axes only for bottom left plot of group (a 'group' is an analysis of identical markers).
- [x] All plots are contour plots with outliers or pseudocolor plots.
- [x] A numerical value for number of cells or percentage (with statistics) is provided.

Methodology

Sample preparation

PBMC isolation
Blood samples were collected at baseline (i.e. immediately prior to DMARD cessation) and at either flare onset or month 6 sustained drug-free remission Healthy control PBMCs (technical biological replicates) were obtained at a single time point from a single healthy donor provided as a cone product by National Health Service Blood and Transplant. Blood was diluted in an equal volume of calcium/magnesium-free Hanks medium (Lonza, catalogue number BE10-543F) with 2mM ethylenediaminetetraacetic acid (EDTA, Thermo-Fisher Scientific, catalogue number BP2482100). Between 15-25ml of diluted blood was then layered onto 15ml of LymphoPrep™ (Axis-Shield Diagnostics, catalogue number NYC 1114547) and spun at 895g for 30 minutes at room temperature with slow acceleration with no brake. PBMCs were recovered from the density interface by pipetting, and suspended in wash medium containing 50ml of calcium/magnesium-free Hanks medium with 1% foetal bovine serum (FBS) (Thermo-Fisher Scientific, catalogue number Gibco 10270-106; Biosera, catalogue number FB-1550/500; Labtech, catalogue number FCS-SA/500) at 4°C. Samples were spun at 600g for 7 minutes at 4°C, the supernatant discarded, and the pellet resuspended in 50ml wash medium at 4°C. Samples were then spun at 250g for 7 minutes at 4°C, the supernatant discarded and the cells resuspended in 10ml of wash medium and passed through a 70µm nylon filter (Greiner bio-one, catalogue number 542070). The cell concentration was measured using a Burker counting chamber, and samples were then spun at 400g for 7 minutes at 4°C. The supernatant was discarded, and the cells resuspended in FBS with 10% Dimethyl sulfoxide (DMSO, Sigma-Aldrich, catalogue number D2650) in 1ml aliquots of between 5-10 million cells and stored overnight at -80°C before transfer to long-term storage at -150°C.

Mass cytometry
A 44-marker pan-PBMC mass cytometry panel was designed, incorporating CD45 barcoding to allow multiplexing of 5 samples within each batch (Supplementary Table 2). Antibodies conjugated to 89-yttrium and 209-bismuth were supplied by the manufacturer (Standard Biotools). In-house conjugation of CD45 antibody with palladium isotopes (Trace Sciences) was performed using the metal ion chelator isothiocyanobenzyl-EDTA (Dojindo, catalogue number 105394-74-9). Metal conjugation of other antibodies was performed using the Maxpar® x8 Multi-Metal Labelling Kit (Standard Biotools, catalogue number 201300) according to the manufacturer's instructions and including additional 113-indium, 115-indium and 157-gadolinium isotopes (Trace Sciences). PBMCs were thawed at 37°C for 5 minutes, then suspended in 20ml of thaw medium containing calcium/magnesium-free Hanks medium (Lonza, catalogue number BE10-543F) with 10% FBS. The samples were then spun at 400g for 8 minutes, the supernatant discarded and the pellet resuspended in 25ml thaw medium at 37°C. The samples were then spun again at 400g for 8 minutes, the supernatant discarded, and the pellet resuspended in 2ml of thaw medium and rested for 1 hour at 37°C. The cell concentration was measured using a Burker counting chamber. Following addition of 23ml of thaw medium, the samples were spun at 400g for 8 minutes, the supernatant discarded, and the pellet resuspended in 200µL thaw medium per 1 million cells per well in a 96-well plate (Griener bio-one, catalogue number 651101). Subsequent wash steps were performed at 500g for 5 minutes at room temperature using wash buffer containing calcium/magnesium-free Dulbecco phosphate-buffer saline (DPBS) (Sigma-Aldrich, catalogue number D8537) with 2% FBS. The cells were washed once, and stained with barcoding CD45 antibodies in wash buffer (total staining volume 50µL per well) for 30 minutes at room temperature. The cells were then washed once, followed by incubation with 50µL DPBS with 2.5µM cisplatin (Standard Biotools, catalogue number 201064) for 5 minutes at room temperature. Cells were then washed twice, and pooled for extracellular antibody mastermix staining in wash buffer (total staining volume 100µL per well) for 60 minutes at room temperature. Cells were washed twice with DPBS, followed by fixation in 100µL eBioscience® working fix buffer (Thermo-Fisher Scientific, catalogue number 00-5523) with 100µL of DPBS with 3.2% formaldehyde (TAAB Laboratories Equipment Ltd, catalogue number F017/3) for 30 minutes at room temperature. Cells were then spun at 500g for 5 minutes, the supernatant discarded, and the cells resuspended in 200µL eBioscience® working perm buffer (Thermo, catalogue number 00-5523). This process was repeated, followed by intracellular antibody mastermix staining in working perm buffer (total staining volume 100µL per well) for 60 minutes at room temperature. The cells were then washed twice in DPBS, and then incubated in 200µL of DPBS with 125nM iridium (Standard Biotools, catalogue number 201192A) and 1.6% formaldehyde for 60 minutes at room temperature. Cells were then washed and stored in 200µL wash buffer overnight at 4°C before acquisition using a CyTOF mass cytometer (Helios, Standard Biotools) at 30 µL/min after calibrating against the manufacturer's tuning protocol. Samples were acquired in 20 batches of 5 samples each - one technical replicate and 4 patient samples (i.e. paired samples from 2 patients per batch).

Single cell RNA sequencing
PBMCs were thawed at 37°C for 5 minutes and then suspended in 20ml of thaw medium. The cells were washed twice in 25ml thaw medium at 400g for 8 minutes, the cell concentration was measured using a Burker counting chamber, and then the cells were resuspended in 200µL thaw medium per 1 million cells per well in a 96-well plate. Subsequent wash steps were performed at 400g for 3 minutes at room temperature using fluorescence-activated cell sorting (FACS) buffer containing calcium/magnesium-free DPBS with 0.5% bovine serum albumin (Sigma-Aldrich, catalogue number A2153), 1mM EDTA (Sigma-Aldrich, catalogue number E7889) and 0.01% sodium azide (Sigma-Aldrich, catalogue number S2002). The cells were washed and then stained with fluorescent antibody mastermix (Supplementary Table 3) in FACS buffer with 200ng polyclonal

human IgG (Octagam, Octapharma Ltd) at 4°C for 30 minutes (final staining volume 50µL) and protected from light. The cells were washed twice, and then incubated with 20µL of Via-Probe (BD Biosciences, catalogue number 555815) at 4°C for 10 minutes and protected from light. The cells were then suspended in 1ml FACS buffer and kept on ice for immediate fluorescence-activated cell sorting (FACS).

Instrument

Mass cytomery samples were acquired using a CyTOF mass cytometer (Helios, Standard Biotools). Fluorescence-actived cell sorting was performed using FACS Fusion and FACS Aria III cell sorters (BD Biosciences)

Software

Further processing and analysis of deconvoluted mass cytometry data was performed in the R statistical environment (v3.4). Normalisation of inter-batch variation was achieved by reference to the technical replicate samples using the Normalizebatch () function of the cydar package (v1.2.1). Further analysis was performed according to the published workflow of Nowicka et al. Unsupervised clustering of normalised data using FlowSOM (version 1.10.0) and ConsensusClusterPlus (v1.42.0) packages was performed, using a 15 x 15 self-organising map followed by reduction to 50 clusters. Manual merging of clusters was performed based upon similarity of canonical lineage marker expression.

Cell population abundance

Three cell populations were isolated by FACS for scRNAseq analyses: CD3+CD4+CD45RO+PD1hi T cells (median(IQR) 13 (9-17)% of total CD4+ T cells), CD3+CD8+CD45RO+PD1hi T cells (median(IQR) 15(13-18)% of total CD8+ T cells), and CD19+ B cells.

Gating strategy

Singlets were first identified (FSC-H vs FSC-A), followed by removal of debris (SSC-A vs FSC-A) and then removal of dead cells (7AAD vs FSC-A) and dump-channel positive cells. Next, CD8 vs CD3 gating was applied to identify CD8+ and CD8- cells. CD8+ cells were further gated by CD3 vs CD4, with CD8+CD3+CD4- cells gated by PD1 vs CD45RO to isolate the target CD3+CD8+CD4-CD45RO+PD1hi T cells. CD8- cells were gated by CD4 vs CD3 to identify CD3+CD8-CD4+ cells, and CD3-CD8-CD4- cells. CD3+CD8-CD4+ cells were then gated by PD1 vs CD45RO to isolate the target CD3+CD8+CD4-CD45RO+PD1hi T cells. CD3-CD8-CD4-/lo cells were then gated by CD19 vs CD3 to isolate the target CD19+ B cells.

☒ Tick this box to confirm that a figure exemplifying the gating strategy is provided in the Supplementary Information.
